# Navigating Cetacean Mitochondrial Genome Data: Identifying Coverage and Deficiencies in Public Repositories

**DOI:** 10.1111/1755-0998.70141

**Published:** 2026-04-16

**Authors:** Luís Afonso, Alessandro Lagrotteria, Ana Sofia Lavrador, Paula Suarez‐Bregua, Miguel Álvarez‐González, Raul Valente, Mads Reinholdt Jensen, Camilo Saavedra, Daniel Kumazawa Morais, Isabel Sousa‐Pinto, Graham J. Pierce, Catarina Magalhães, Ana Mafalda Correia

**Affiliations:** ^1^ Interdisciplinary Centre of Marine and Environmental Research (CIIMAR/CIIMAR‐LA) University of Porto Matosinhos Portugal; ^2^ School of Medicine and Biomedical Sciences (ICBAS‐UP) University of Porto Porto Portugal; ^3^ Institute of Marine Research, Spanish National Research Council (IIM‐CSIC) Vigo Spain; ^4^ Research Institute on Terrestrial Ecosystems Italian National Research Council (CNR‐IRET) Florence Italy; ^5^ Department of Life Sciences and Systems Biology University of Turin Turin Italy; ^6^ Oceanographic Centre of Vigo, Spanish National Research Council (IEO‐CSIC) Vigo Spain; ^7^ Biology Department of the Faculty of Sciences (FCUP) University of Porto Porto Portugal; ^8^ Norwegian College of Fishery Science UiT—The Arctic University of Norway Tromsø Norway

**Keywords:** BOLD, data repositories, environmental DNA, gap analysis, GenBank, marine mammals, mitogenome

## Abstract

Genetic reference databases underpin a wide range of molecular approaches used to study cetacean biodiversity, including environmental DNA (eDNA), yet their reliability depends critically on data completeness, taxonomic accuracy, and metadata quality. Here, we present the first global assessment of mitochondrial sequence availability for cetaceans, evaluating taxonomic coverage, geographic representation, metadata completeness, and the distribution of five commonly targeted mitochondrial markers (12S rRNA, 16S rRNA, D‐loop, cytochrome oxidase I, and cytochrome b). We retrieved 17,569 cetacean accessions from the NCBI Nucleotide database and an additional 259 COI‐only records from BOLD Systems. Sequence availability was strongly biased toward Delphinidae and Balaenopteridae, whereas several families, notably Ziphiidae, were markedly underrepresented. We also identified discrepancies between database records and currently accepted cetacean taxonomy (e.g., outdated genera, non‐accepted species, and collapsed higher‐level taxa). Among markers, the D‐loop dominated database representation, largely as standalone sequences (12,437 records), reflecting historical and current sequencing priorities and underscoring its continued relevance for population‐level studies and eDNA marker development. Only 38% of accessions included geographic metadata, with georeferenced records concentrated primarily in the Americas and the Northwest Pacific, while large regions, including much of Africa, remained poorly represented. Although broad geographic patterns mirrored known family distributions, pronounced regional and taxonomic gaps persist. Our results highlight critical deficiencies in mitochondrial reference databases for cetaceans and emphasise the need for improved metadata standards, targeted sequencing of underrepresented taxa and regions, and open data sharing to enhance the effectiveness and global applicability of eDNA‐based cetacean monitoring.

## 1 Introduction

Cetacea, an infraorder of marine mammals that includes whales, dolphins, and porpoises, has extraordinary ecological and evolutionary relevance. These animals occupy diverse ecological niches, contribute to marine ecosystem structure and function, and exhibit distinct physiological and behavioural adaptations to aquatic life (Kiszka et al. 2015, 2022; Roman and Estes 2018). Studying cetaceans presents unique challenges: many species are long‐lived, migratory, deep‐diving, and/or inhabit offshore or remote habitats, making direct observation difficult (Evans and Hammond 2004). Traditional monitoring approaches, such as visual surveys and stranding records, provide valuable insights but are constrained by logistical, temporal, environmental, and financial limitations. Genetic analytical approaches have therefore expanded the scope of cetacean research, enabling high‐resolution investigations of distribution, diversity, population structure, connectivity, and evolutionary history that are difficult to achieve through observation alone (Rosel et al. 2017; Suarez‐Bregua et al. 2022). These molecular tools have reshaped how cetacean populations are detected, characterised, and monitored, making the availability and quality of genetic reference resources critical to modern research. Understanding their strengths and limitations is essential to ensure reliable application and interpretation across cetacean research contexts.

Recent advances in high‐throughput sequencing have further expanded these possibilities. Techniques such as eDNA metabarcoding enable species detection and biodiversity assessment directly from environmental samples, including water, soil, or air, without requiring visual confirmation (Gold et al. 2022). These approaches are particularly promising for cetaceans, which are often elusive or sparsely distributed. However, their reliability depends on comprehensive, well‐curated reference databases, as accurate taxonomic assignment requires matching environmental sequences to confidently annotated records (Blackman et al. 2023; Keck et al. 2023). This limitation also extends to molecular assay development, as designing effective primers and markers, particularly for genetically structured or habitat‐fragmented populations, requires reference sequences that adequately represent the genetic diversity and geographic origins of target taxa (Wilcox et al. 2013). While local repositories may support regional studies, most eDNA research relies on large public databases, notably the NCBI Nucleotide database and the Barcode of Life Data (BOLD) Systems, that provide global taxonomic frameworks (Blackman et al. 2023; Fleming 2023). Insufficient, biased, or incorrectly annotated reference data can produce uncertain identifications and limit biodiversity assessments (Teixeira et al. 2025). Evidence from fish, annelids, and marine macroinvertebrates shows that taxonomic and geographic gaps in reference databases can compromise eDNA monitoring (Claver et al. 2023; Teixeira et al. 2025; Vieira et al. 2021), underscoring the need to evaluate sequence availability across target taxa such as cetaceans.

Mitochondrial DNA (mtDNA) has long played a central role in cetacean research (Ohland et al. 1995). Its small size, maternal inheritance, and high mutation rate make it effective for species identification, phylogenetic inference, and population studies (Alexandre et al. 2024; Lee et al. 2019; Sasaki et al. 2005). Over the past three decades, thousands of mitochondrial sequences have accumulated in nucleotide databases (Smith 2016), largely driven by the increasing whole‐genome sequencing efforts (Leigh et al. 2021), forming a foundation for traditional genetics and emerging eDNA applications. 12S rRNA (12S; Ushio et al. 2025; Valsecchi et al. 2020), and 16S rRNA (16S; Valsecchi et al. 2020)—to moderately variable protein‐coding genes—cytochrome oxidase I (COI; Robinson et al. 2024) and cytochrome b (Cytb; López‐Oceja et al. 2019; Parsons et al. 2018)—and the highly variable mitochondrial control region (D‐loop; Parsons et al. 2018; Parsons et al. 2025). However, the completeness, taxonomic distribution, and metadata quality of these public sequences have not been systematically evaluated. Identifying coverage gaps is essential for assessing molecular tool reliability and ensuring robust eDNA monitoring outcomes.

Documenting the diversity and distribution of cetacean mitochondrial genomes in public databases is therefore critical to strengthening the molecular foundation of cetacean research and conservation. This study presents the first global assessment of these resources, focusing mainly on the NCBI Nucleotide database. We evaluate gaps in taxonomy, metadata completeness, and geographic coverage of mitochondrial markers, highlighting uneven representation across cetacean taxa. By identifying these limitations, the analysis establishes a framework for improving the reliability and interpretability of genetic tools across applications, including population genetics, biodiversity assessment, and eDNA monitoring, ultimately supporting stronger ecological research, conservation, and management planning.

## 2 Methods

### 2.1 Data Extraction, Marker Representation, and Co‐Occurrence

Five mitochondrial markers were selected: 12S rRNA, 16S rRNA, COI, Cytb, and the D‐loop, all widely used in wildlife eDNA metabarcoding studies (Othman et al. 2021). These markers capture complementary levels of mitochondrial genetic variability, supporting taxonomic identification in cetacean‐targeted analyses. The ribosomal genes 12S and 16S are relatively conserved, enabling robust amplification across taxa and reliable higher‐level assignments (Ushio et al. 2025; Valsecchi et al. 2020). In contrast, the faster‐evolving protein‐coding genes COI and Cytb provide greater species‐level resolution (Robinson et al. 2024; Ma et al. 2016). The mitochondrial control region (D‐loop), the most variable part of the genome, is commonly used to resolve population structure and fine‐scale evolutionary processes in cetaceans (Baker et al. 2018; Parsons et al. 2018). Sequence information available in the NCBI Nucleotide Database (https://www.ncbi.nlm.nih.gov/; last assessment conducted on 04/07/2025) for all these markers was extracted in R (v4.5.0) via RStudio, using the *rentrez* package (v1.2.3; Winter 2017), following the queries described in Table 1. All queries included both gene‐specific keywords and broader terms (“complete genome” and “mitochondrial genome”) to ensure the retrieval of complete mitochondrial genomes containing the target markers. Because gene annotations in public repositories are not always standardised (e.g., mitochondrial COI sequences variably labelled as COI, CO1, COXI, or COX1), queries were designed to account for known annotation variants to minimise incomplete retrieval.

**TABLE 1:** Data extraction query and inclusion keywords for database filtering for each of the target mitochondrial markers in the NCBI Nucleotide database (last assessed on 04/07/2025). COI—Cytochrome c oxidase subunit I; 12S—12S ribosomal RNA; 16S—16S ribosomal RNA; Cytb—Cytochrome b; D‐loop—Control region.

Marker	NCBI extraction query	Inclusion keywords
COI	Cetacea[Organism] AND (COI[Gene] OR CO1[Gene] OR COXI[Gene] OR COX1[Gene] OR “complete genome”[All Fields] OR “mitochondrial genome”[All Fields])	cytochrome c oxidase subunit I; cytochrome c oxidase subunit 1; cytochrome oxidase subunit I; cytochrome oxidase subunit 1; CO1; COI; COX1
12S	Cetacea[Organism] AND (“12S”[All Fields] OR “12S ribosomal RNA”[Title] OR “12S rRNA”[Title] OR “complete genome”[All Fields] OR “mitochondrial genome”[All Fields])	12S ribosomal RNA; 12S ribosonal RNA; 12S small subunit ribosomal RNA; small subunit ribosomal RNA; s‐rRNA
16S	‘Cetacea[Organism] AND (“16S”[All Fields] OR “16S ribosomal RNA”[Title] OR “16S rRNA”[Title] OR “complete genome”[All Fields] OR “mitochondrial genome”[All Fields])	16S ribosomal RNA; large subunit ribosomal RNA; l‐rRNA
Cytb	Cetacea[Organism] AND (CYTB[Gene] OR “cytochrome b”[Gene] OR “cyt b”[All Fields] OR “complete genome”[All Fields] OR “mitochondrial genome”[All Fields])	CYTB; cytochrome b
D‐loop	Cetacea[Organism] AND (“D‐loop”[All Fields] OR “control region”[All Fields] OR “complete genome”[All Fields] OR “mitochondrial genome”[All Fields])	D‐loop; control region; D‐loop partial sequence; mitochondrial control region; mitochondrial DNA control region

Following retrieval, NCBI flatfiles were parsed in R and records were filtered to remove non‐target sequences using NCBI feature annotations and associated qualifiers. For each record, the accession number, organism name, taxonomic classification, and bibliographic information was extracted. Each feature was stored with its feature key (“feature type”) and genomic location, and qualifiers were extracted when provided in the format/qualifier = “value” (e.g., gene, product, note). Marker‐specific filtering was then applied to retain only entries explicitly annotated for the target mitochondrial marker through standardised keyword (Table 1) matching in the “feature type,” “gene,” “product,” and “note” fields. Features annotated with unrelated genes, ambiguous descriptors, or lacking explicit marker identification were discarded. Extracted metadata included accession number, feature type, genomic annotation, organism name, taxonomic classification, bibliographic fields, and any source qualifiers captured in the record (e.g., geographic descriptors where available). Filtered records were compiled into the final dataset (Table S1). A unique accession was defined as a record containing an accession number and assigned species, regardless of the number of target markers present.

For comparison, COI records (COI and COI‐5P) were extracted from BOLD Systems (https://boldsystems.org/; last assessment on 4th July 2025) using all accepted cetacean species listed in WoRMS (https://www.marinespecies.org/; accessed 25 April 2025). The COI dataset included sequences annotated as cytochrome oxidase I without restriction on fragment position or length, whereas COI‐5P represents the standardised ~650 bp 5′ barcode fragment. Including both categories ensured capture of standardised barcodes and non‐standard COI fragments. Data were retrieved using the *bold* R package (v1.3.0; Dubois and Chamberlain 2025), and records mined from NCBI (labelled in the “institution_storing” field as “Mined from Genbank”) were removed to retain only native BOLD entries.

All custom R scripts used for data extraction, filtering, and compilation are openly available in the project's GitHub repository ([https://github.com/asdcl/Cetacean‐mitochondrial genome‐gap‐analysis/]). The final curated dataset has been deposited as (Table S1) and archived in Zenodo (https://zenodo.org/records/17733928).

To represent the proportional abundance of sequences for each mitochondrial marker at both the family and species levels, summary tables were generated from the final curated dataset using R. For each taxonomic rank, the total number of unique accessions per marker was calculated. Data for all marker‐taxon combinations were visualised as barplots using the *ggplot2* package (v3.5.2; Wickham, Chang, and Wickham 2016).

For each unique NCBI accession, the presence or absence of five mitochondrial markers (D‐loop, Cytb, COI, 16S, and 12S) was recorded. A marker was considered present if any portion of the corresponding gene region was annotated or clearly identifiable within the sequence, regardless of length or completeness. Thus, even short or partial fragments were counted to capture all available mitochondrial information and avoid underestimating marker representation. Markers were considered absent when no annotated or identifiable sequence corresponding to the gene was detected. To assess temporal trends in data production, the publication year was extracted for records associated with a scientific output. Publication year was used rather than sequence submission date because it provides a more consistent and reliable temporal reference, reflecting when the study was conducted rather than when data were deposited. The resulting binary matrix of marker combinations was summarised and visualised using an UpSet plot generated with the *ComplexUpset* package (v1.3.3; Krassowski, 2020) and *ggplot2* in R. Temporal patterns were additionally explored using a matrix plot created with the *scales* (v1.4.0; Wickham et al. 2016) and *forcats* (v1.0.1; Wickham and Wickham 2021) packages.

### 2.2 Metadata Completeness and Geographic Coverage

The completeness of geographic metadata associated with cetacean mitochondrial sequences was assessed by compiling all accessions retrieved from NCBI into a single database. Sequences were classified according to their publication status (published versus unpublished) and the availability of geographic information. Records were classified as published when accession metadata included an associated peer‐reviewed journal reference in the NCBI “Journal” fields, whereas records lacking such references were treated as unpublished. Records with submission information or associated with a thesis were also considered as published for this analysis. Geographic metadata were evaluated based on the presence of the geo_loc_name field (location name), lat_lon qualifier (geographic coordinates), or both. A sequence was considered to contain geographic information if either the geo_loc_name field or any coordinate qualifier included a valid entry, regardless of the level of spatial precision (e.g., ocean basin, country, or specific locality). Entries containing partial information, such as a country name without coordinates, were classified as having geographic metadata. Metadata completeness was visualised using a Sankey diagram, produced in R with the *networkD3* package (v0.4.1; Allaire et al. 2025).

To further assess the geographic coverage, all NCBI's accessions containing spatial metadata in the aforementioned fields were georeferenced. When both types of geographical information were available, coordinates were verified to determine the exact sample location. For sequences containing only *geo_loc_name* data, a specific locality was assigned when available, or a corresponding country when the exact site was not defined. For sequences with coordinates but no country designation, geographic locations were automatically retrieved using the *rnaturalearth* package in R (v1.1.0; Massicotte and South 2025).

To standardise global record distribution and reduce spatial heterogeneity, sequences were aggregated into 40 predefined geographic clusters representing major oceanic regions and locally relevant marine basins while minimising record fragmentation. The Atlantic, Pacific, and Indian Oceans were subdivided into clusters based on cardinal and intercardinal directions. Additional clusters were defined for the Arctic Ocean (American, European, and Asian sectors) and Antarctica (treated as a single cluster). To simplify the scheme, some gulfs were merged with adjacent oceanic regions. The Gulf of Alaska was incorporated into the North Pacific Ocean cluster, and the Gulf of Mexico was combined with the Caribbean Sea to form a Central America–Atlantic Ocean cluster. Two isolated seas—the Mediterranean Sea and the Black Sea—were retained as distinct clusters due to their separation from the major oceans. Clusters were classified as “Unknown” (e.g., USA Unknown) when sequences lacked coordinates and were georeferenced only by country (geo_loc_name), where coastlines span multiple marine regions and prevent specific assignment. Representative coordinates were assigned to each cluster to enable spatial plotting and visualisation; for “Unknown” clusters, coordinates corresponded to the centroid of the associated country. Cluster attribution for each accession, based on retrieved geographic metadata, is provided in Table S1. These clusters were used to generate visualisation maps in ArcGIS Pro, showing the number of unique accessions and cumulative marker sequences per region, along with marker proportions and the taxonomic distribution of records by cetacean family, providing a global framework for assessing geographic coverage.

### 2.3 Database Comparison (NCBI vs. BOLD Systems)

To compare different public data repositories, COI datasets retrieved from NCBI and BOLD were matched. COI and COI‐5P datasets were compiled, and duplicates were removed. Sequences were then grouped at the family level, and the total number of sequences available for each family was calculated in both datasets. The comparative analysis of sequence availability between the NCBI and BOLD was summarised in a bar plot generated in R using the *ggplot2* package.

## 3 Results

### 3.1 Data Extraction, Marker Representation, and Co‐Occurrence

In total, 27,908 records were extracted and filtered for analysis from the NCBI Nucleotide, corresponding to 17,569 unique accession numbers containing at least one of the studied markers (Table S1). Among the five mitochondrial markers analysed from NCBI, the D‐loop was the most frequent, with a total of 15,001 records across all cetacean species. This was followed by cytochrome b (Cytb) and cytochrome oxidase I (COI), with 3854 and 3518 records, respectively. The 16S rRNA gene appeared in 2754 accession numbers, while the 12S rRNA had 2700 results.

Summaries of NCBI‐mined records per cetacean family are presented in Table 2, and species‐level information is available in (Table S2). In addition, 259 records were extracted from the COI and COI5P databases of BOLD Systems (Table S3).

**TABLE 2:** . Summary of cetacean family representation in the NCBI Nucleotide database (as of 4 July 2025). The table reports, for each family, the number of unique accession records, the average number of records per valid species (calculated using species counts from the NCBI Taxonomy; https://www.ncbi.nlm.nih.gov/taxonomy), and the average number of records across the five most represented species within that family, calculated at the species level. Under this classification, all sperm whales are included within Physeteridae, encompassing species currently recognised as Kogiidae; consequently, Kogiidae is not shown as a separate category. Families are ordered by total accession count (highest to lowest).

Cetacean family	# unique accessions	# NCBI valid species	Average number of records/species	Average number of records/top 5 species
Delphinidae	8258	42	196.6	890
Balaenopteridae	3656	10	365.6	650,6
Phocoenidae	1237	8	154.6	217,8
Monodontidae	963	2	481.5	NA
Balaenidae	898	4	224.5	NA
Physeteridae	682	3	227.3	NA
Iniidae	653	4	163.3	NA
Ziphiidae	563	24	23.5	77,6
Eschrichtiidae	480	1	480.0	NA
Pontoporiidae	84	1	84.0	NA
Platanistidae	65	2	28.0	NA
Lipotidae	25	1	25.0	NA
Neobalaenidae	5	1	5.0	NA
Total	17,569	103	—	—

Family‐level assignments follow the NCBI taxonomy. Under this classification, all sperm whales are included within Physeteridae, which encompasses Kogiidae species, explaining the absence of a separate Kogiidae category in the results.

**FIGURE 1:**
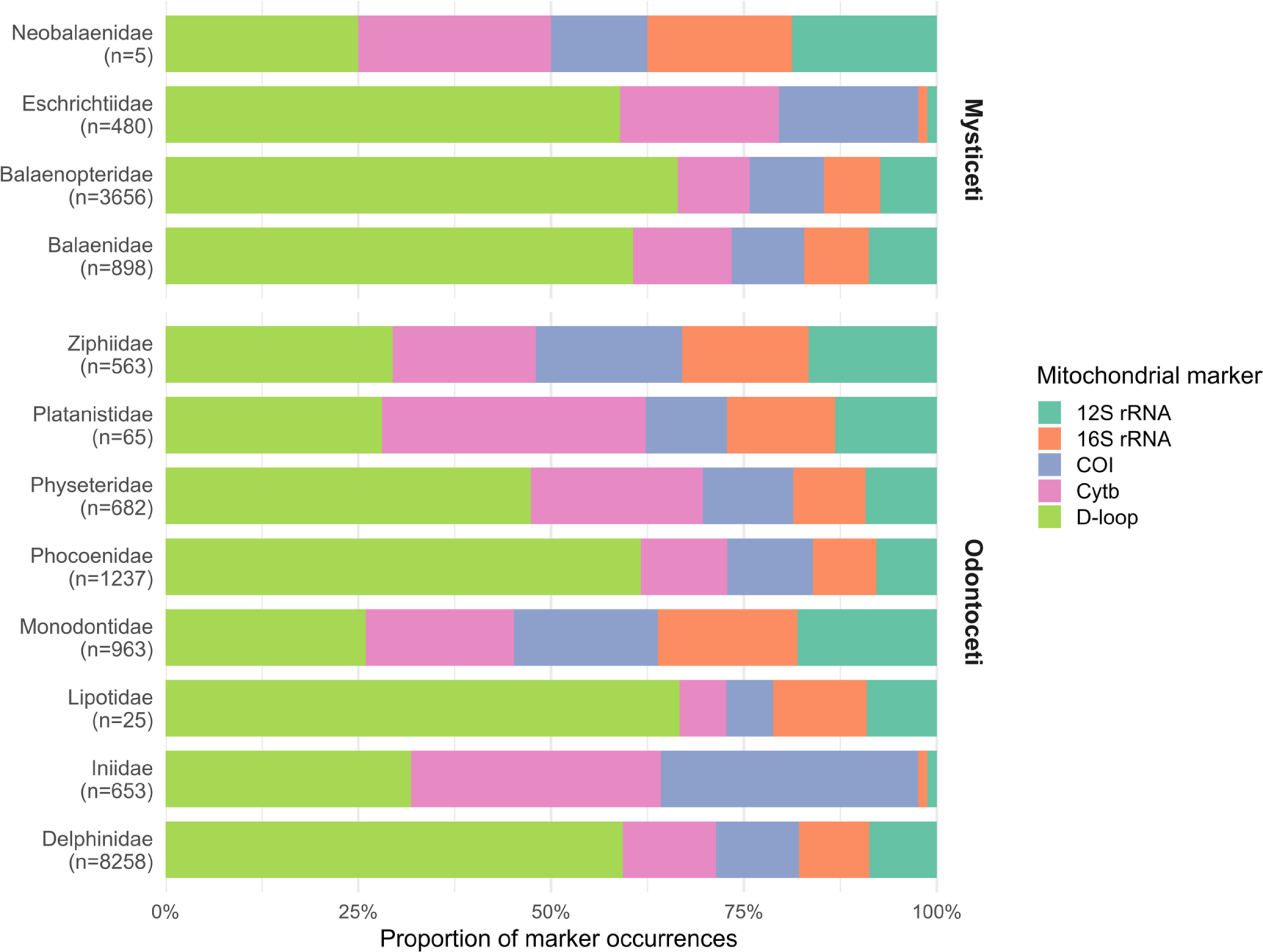
Proportional composition of mitochondrial marker representation across cetacean families, based on records retrieved from the NCBI Nucleotide database (as of 4 July 2025). The five markers (12S rRNA, 16S rRNA, COI, Cytb, and D‐loop) are shown as stacked proportions representing the relative contribution of each marker within families, allowing comparison independent of total sequence abundance. Families are grouped by suborder (Mysticeti and Odontoceti), and the total number of unique accessions per family is indicated as n in the axis labels. Analysis follows NCBI Taxonomy, under which sperm whale species currently classified as Kogiidae are included within Physeteridae and therefore are not shown as a separate family.

Marker proportional sequences per cetacean family are shown in Figure 1. The Delphinidae family (suborder Odontoceti) represented the largest share of unique accessions, with 8258 unique accessions. The vast majority contained D‐loop sequences (7221 records ‐ 87.4%). The remaining markers were represented as follows: Cytb—1467 records (17.8%); COI—1312 records (15.9%); 16S—1112 records (13.5%); 12S—1062 records (12.9%). Among the other Odontocetes, the Phocoenidae family had 1237 unique accessions, of which 1103 corresponded to D‐loop (89.2%), 200 to Cytb records (16.2%), 199 to COI (16%), 147 to 16S (11.9%), and 140 to 12S (11.3%). The Monodontidae family had 963 unique accessions, including 892 D‐loop records (92.6%), 660 Cytb (68.5%), 640 COI (66.5%), 622 of 16S (64.6%), and 620 of 12S (64.4%). These were followed by the Physeteridae and Ziphiidae and families, with 682 and 563 unique accessions, respectively. For beaked whales (Ziphiidae), 441 records corresponded to D‐loop (78.3%), 278 to Cytb (49.4%), 284 to COI (50.4%), 245 to 16S (45.1%), and 249 to 12S (44.2%). For sperm whales (Physeteridae), 497 records corresponded to D‐loop (72.9%), 234 to Cytb (34.2%), 123 to COI (18%), 98 to 16S (14.4%), and 97 to 12S (14.2%). Among river dolphin families, Iniidae accounted for 653 unique accessions, including 221 D‐loop (33.8%), 215 Cytb (32.9%), 221 COI (33.8%), and 8 records each for 16S and 12S (1.2%). The remaining river dolphin families—Platanistidae, Pontoporiidae, and Lipotidae—collectively accounted for a total of 174 unique accessions, including 121 D‐loop (69.5%), 43 Cytb (24.7%), 27 COI (15.5%), 25 16S (14.4%), and 22 12S (12.6%).

Within the suborder Mysticeti, the Balaenopteridae family was the most prominent, representing 3656 unique accessions, of which 3396 contained D‐loop records (92.9%), 477 Cytb (13%), 493 COI (13.5%), and 374 each for 16S and 12S (10.2%). The Balaenidae family followed, with 898 unique accessions, including 821 containing the D‐loop (91.4%), 173 Cytb (19.3%), 127 COI (14.1%), 114 16S (12.7%), and 119 12S (14.5%). The Eschrichtiidae family had 480 unique accessions, including 294 D‐loop (61.3%), 103 Cytb (21.5%), 90 COI (18.8%), and 6 each for 16S and 12S (1.3%). Finally, the Neobalaenidae family had only 5 unique accessions, the smallest number in this study, from which 4 each containing the D‐loop and Cytb (80%), 2 containing COI (40%), and 3 each containing the 16S and 12S (60%).

Overall, the distribution of markers was highly uneven, with the D‐loop accounting for more than half of all sequences, while ribosomal genes remained consistently underrepresented across most families. Odontocetes, particularly Delphinidae, dominate the dataset, whereas river dolphins and several baleen whale families exhibited markedly lower coverage. Figures for all unique taxonomic entries are provided in the (Figure S1).

At the species level, mitochondrial reference coverage was generally high across Cetacea, with most species represented by sequences for all five target markers (12S rRNA, 16S rRNA, COI, Cytb, and D‐loop) (Table S2). Complete multi‐marker coverage was observed for the majority of baleen whales and many delphinids, as well as representatives of Monodontidae, Phocoenidae, Physeteridae, and other families. Nevertheless, several species, particularly within Delphinidae and Ziphiidae, showed partial marker representation, often lacking ribosomal or protein‐coding loci despite the presence of D‐loop sequences. Total sequence counts were highly uneven among species, ranging from single‐digit records in some beaked whales to more than a thousand sequences for widely studied taxa such as *Tursiops truncatus* , *Delphinus delphis* , and *Megaptera novaeangliae* . These differences highlight that, although species‐level representation is broadly complete, marker coverage and sequencing depth remain heterogeneous across taxa.

**FIGURE 2:**
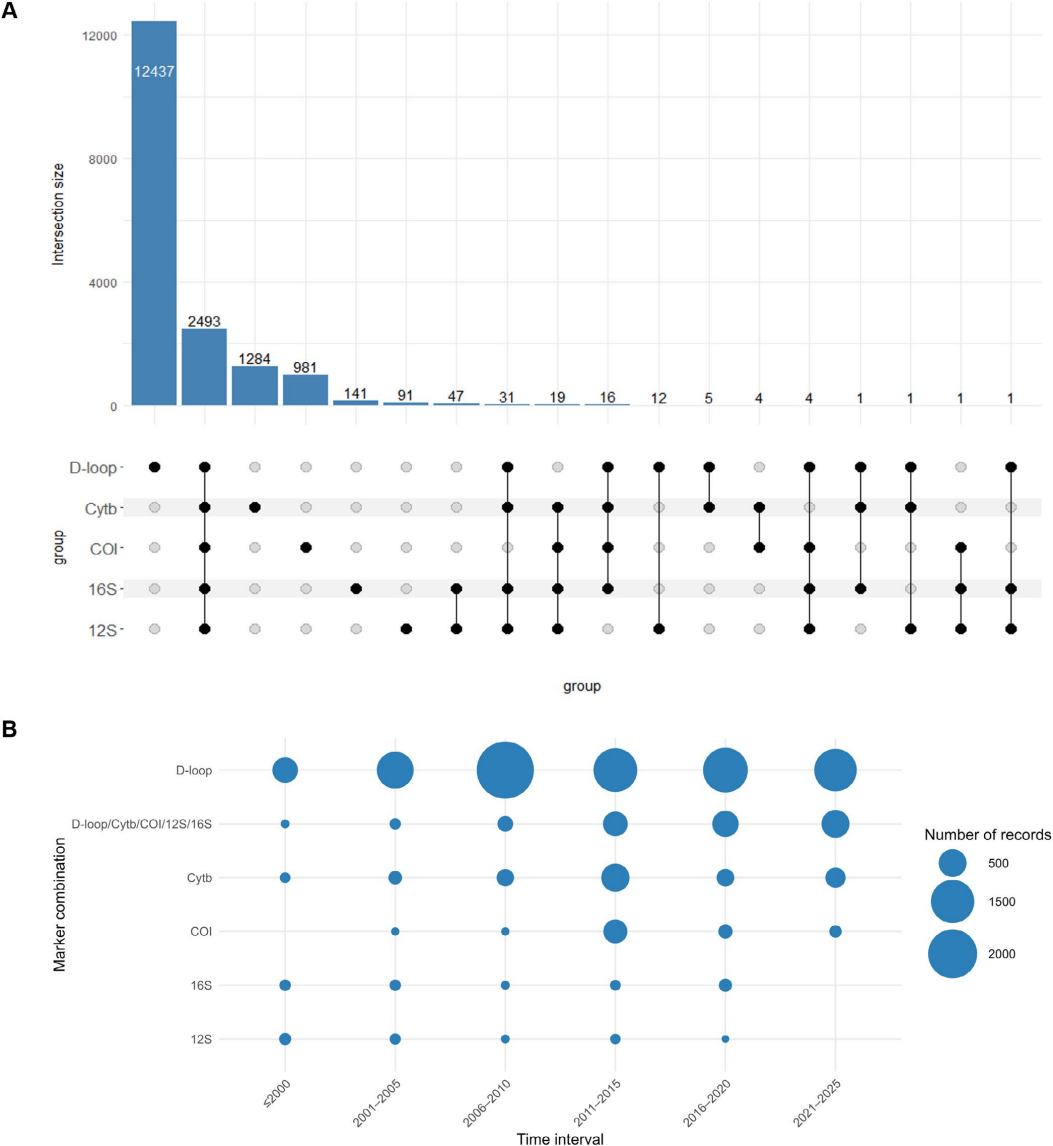
Co‐occurrence of mitochondrial markers (D‐loop, Cytb, COI, 12S and 16S) across cetacean extracted records from the NBCI Nucleotide database (as of July 4, 2025). (A) Total number of unique accessions containing each combination of the five target mitochondrial markers (D‐loop, Cytb, COI, 16S, and 12S). Filled circles indicate marker presence in each combination; (B) Temporal trends across different time intervals (≤ 2000, 2001–2005, 2006–2010, 2011–2015, 2016–2020, 2021–2025) in the most abundant combinations (D‐loop, D‐loop/Cytb/COI/16S/12S, Cytb, COI, 16S and 12S) for records with associated publication/submission year.

Regarding the co‐occurrence of mitochondrial markers across all cetacean data (Figure 2A), 12,437 of the unique accessions contained only the D‐loop marker, representing 70.8% of all extracted records. The second most frequent category was the simultaneous presence of all five markers, observed in 2493 unique accessions (14.2%). This was followed by isolated Cytb sequences (1284; 7.3%), isolated COI sequences (981; 5.6%), isolated 16S sequences (141; 0.8%), and isolated 12S sequences (91; 0.5%). The remaining 126 accessions (0.7%) correspond to partial multi‐marker combinations. Overall, 14,934 accessions (85%) contained only a single marker, whereas 2635 accessions (15%) provided multi‐marker coverage. Temporal analysis of published records (Figure 2B) reveals that isolated D‐loop sequences have been consistently targeted across all time periods, peaking between 2006 and 2010 with a total of 2842 records. The combination of all mitochondrial markers has shown a steady increase over time, reaching its maximum in the most recent interval (2021–2025) with 505 records. In contrast, isolated Cytb and COI sequences were predominantly published between 2011 and 2015 (518 and 336 records, respectively). For the ribosomal genes (12S and 16S), the publication of isolated sequences has remained relatively stable throughout the years, although no records had been published between 2021 and 2025 as of the date of data retrieval.

### 3.2 Metadata Completeness and Geographic Coverage

**FIGURE 3:**
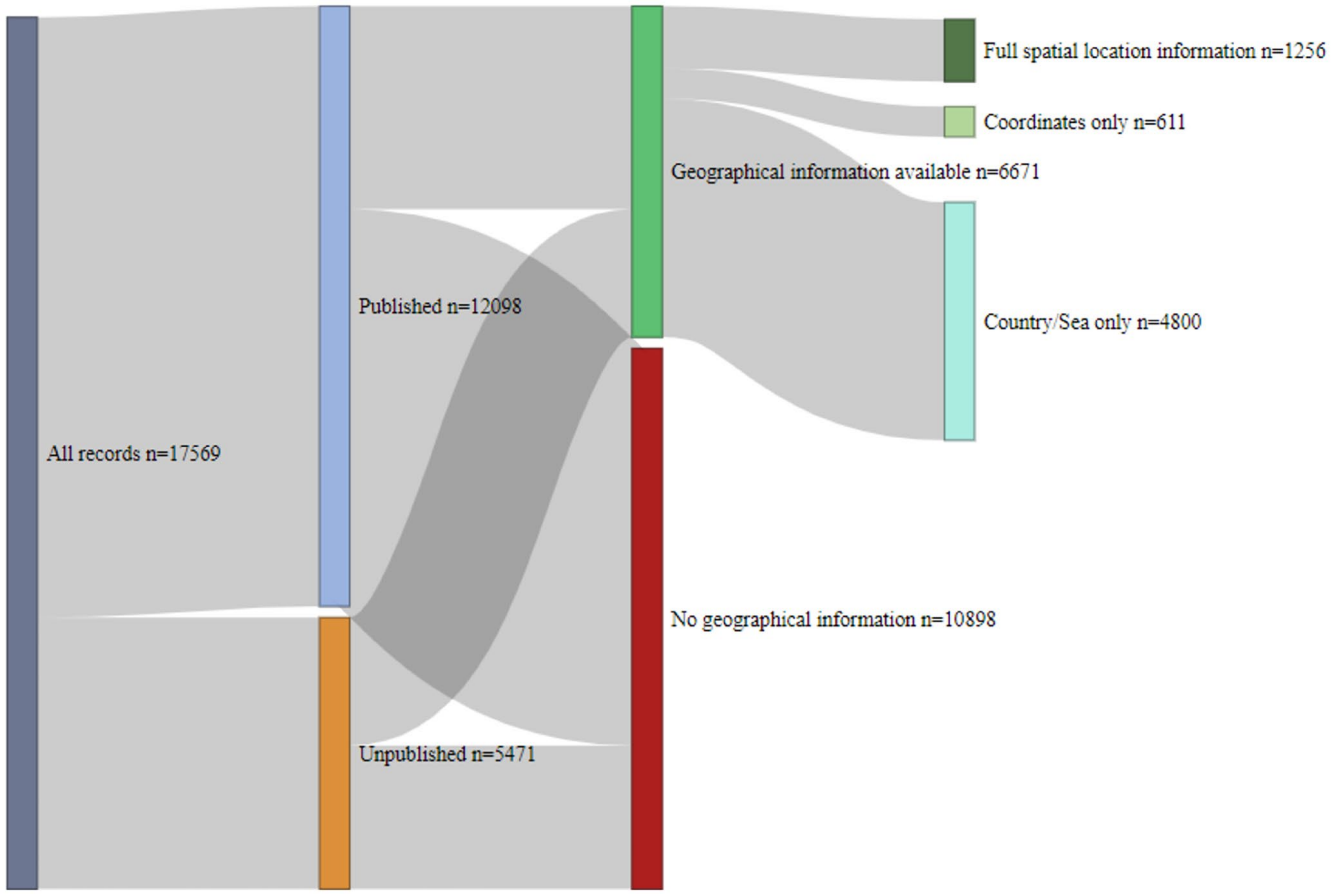
Metadata completeness (geographical information and publication status) of all unique cetacean mitochondrial accessions, retrieved from NCBI Nucleotide database (assessed on 4th of July 2025). “Country/Sea only” refers to records containing only the “geo_loc_name” (reported sampling location associated with each accession), which may be provided in addition to or instead of coordinates.

To assess the availability of geographic metadata for cetacean mitochondrial sequences, we examined whether each accession included location information and whether it was linked to a publication (Figure 3). Of the 17,569 unique accessions extracted from NCBI, 6671 (38%) contained geographic information in at least one of the relevant metadata fields. Among these, 4800 accessions (27.3%) had a country and/or region specified in the “geo_loc_name” field, 611 (3.5%) provided only coordinates, and 1256 (7.2%) included both—representing complete geographic metadata. Among accessions with geographic data, 4092 (61.3%) were linked to scientific publications, while 2579 (38.7%) were not. Conversely, of the 10,898 accessions (62.0%) lacking geographic information, 8006 (45.5%) were associated with publications, and 2892 (16.5%) were unpublished. Overall, geographic information was present for a minority of accessions, indicating that the majority of records lack spatial data regardless of publication status.

**FIGURE 4:**
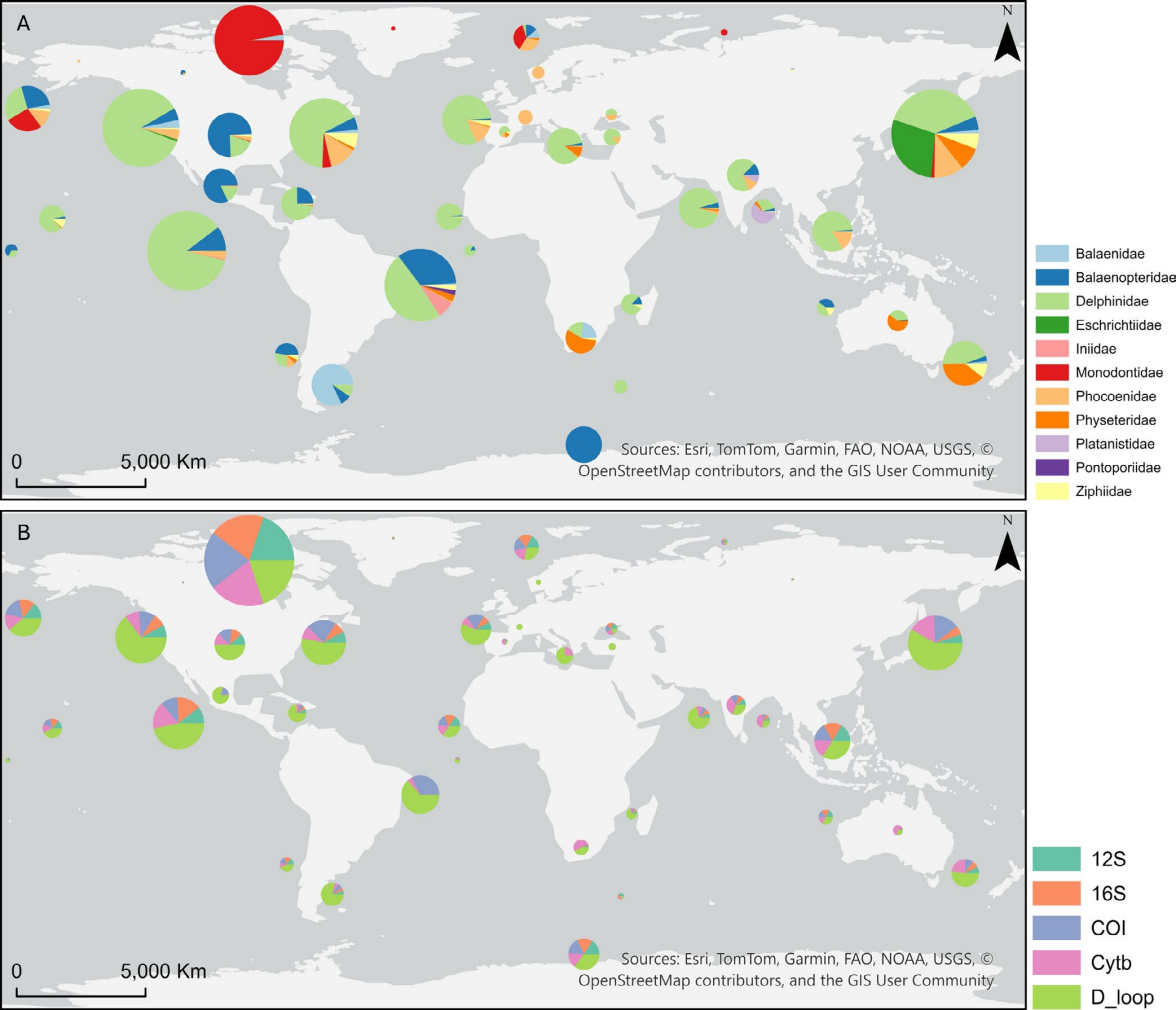
Geographic distribution of cetacean mitochondrial sequences retrieved from NCBI Nucleotide database (assessed on 4th July 2025). (A) Distribution of unique accession numbers per cetacean family across the 40 geographic clusters. Pie chart size is proportional to the number of accessions in each geographical cluster in comparison with the others, and sector size reflects the relative contribution of each cetacean family to its cluster. (B) Cumulative sequences of the five target mitochondrial markers (12S rRNA—12S, 16S rRNA—16S, cytochrome c oxidase I—COI, cytochrome b—Cytb, and control region—D‐loop) across 40 geographic clusters, where cumulative sequences represent the sum of all marker occurrences within a cluster. Pie chart size is proportional to the total number of cumulative sequences in a geographical cluster in comparison with the others, and sector size reflects the relative contribution of each marker to its cluster. Pie charts of countries' “Unknown” clusters were positioned in central coordinates for the given country.

In relation to the geographical distribution of accessions containing location metadata, the Northwest Pacific Ocean led with 844 unique accessions (Figure 4A), followed by East Central Pacific (687) and Northeast Pacific (658). These were followed by the West Central Atlantic Ocean (571), the American Arctic Ocean (532), and the Northwest Atlantic (520). All remaining clusters contained fewer than 300 unique accessions. At the lower end of representation, excluding countries' “Unknown” clusters, are the West Indian Ocean (47 unique accessions), East Indian Ocean (33), Southwest Indian Ocean (20), Black Sea (15), and Asian Arctic Ocean (5 unique accessions), which showed the poorest coverage.

Looking at the geographic distribution of unique accessions across cetacean families (Figure 4A, Table S4), clear taxonomic patterns emerge, as might be expected given the known geographic distributions (and relative abundances) of the various families. The Delphinidae family dominates tropical, subtropical, and temperate clusters worldwide. The largest numbers of accessions are found in the East Central Pacific (591), Northeast Pacific (565), and Northwest Atlantic Ocean (348). Delphinidae are absent from only eight of the 40 geographic clusters. Monodontidae records are concentrated across eight Arctic clusters, with the American Arctic Ocean alone comprising 518 unique accessions. Among other odontocetes, Phocoenidae are represented in 22 geoclusters, with the highest incidence in the Northwest Pacific Ocean (93 accessions), and notable contributions in the Northwest Atlantic (68). The Ziphiidae and Physeteridae families display similar patterns, being present in 23 and 16 clusters, respectively, with overlap in 14 clusters ranging from temperate to tropical and subtropical regions. Ziphiidae reach their highest representation in the Northwest Pacific Ocean (48 accession numbers), followed by the Northwest Atlantic Ocean (37). Physeteridae records peak in the Southwest Pacific (85 unique accessions) and in the Northwest Pacific (72). As expected, river dolphin families are much less represented: Iniidae and Platanistidae appear in only two clusters each, Pontoporiidae in one, while Lipotidae has no records with geographic metadata.

Within the Mysticeti, the Balaenopteridae show the broadest geographical distribution of accessions, with marked representation in the West Central Atlantic Ocean (197 unique accession numbers), USA Unknown (160), and Antarctica (146). This family occurs in 28 of the 40 clusters, though representation is minimal in several regions, including European‐associated clusters, such as Northeast Atlantic Ocean (3 accessions) and the Mediterranean Sea Basin (4), African Atlantic Coast such as East Central Atlantic (1), and West/Central Pacific clusters (1 accession each). Balaenidae are present in 12 clusters, mostly in temperate and polar regions, with a strong concentration in the Southwest Atlantic Ocean (157; around 60.1% of the cluster's total). Eschrichtiidae appear in only 2 clusters, with 97.6% of sequences (242) concentrated in the Northwest Pacific Ocean. Neobalanidae are absent due to a lack of geographic metadata.

When examining the cumulative number of mitochondrial marker sequences across clusters (Figure 4B, Table S5), the American Arctic Ocean stands out with 2576 sequences, showing a strikingly balanced distribution across genes (532 COI and 511 for each of the other markers). This is followed by the Northwest Pacific Ocean with 1066 sequences (620 D‐loop, 172 Cytb, 164 COI, 56 16S, and 54 12S). In general, almost all clusters are dominated by D‐loop sequences, which account for more than or close to 50% of cumulative marker sequences. The lowest cumulative sequence counts, again excluding countries' unknown clusters, are in the Asian Arctic Ocean and the Southwest Indian Ocean (25 cumulative marker sequences).

Maps representing the cumulative marker abundance at the family level across geographical clusters are provided in the (Figure S2).

### 3.3 Database Comparison (NCBI vs. BOLD Systems)

**FIGURE 5:**
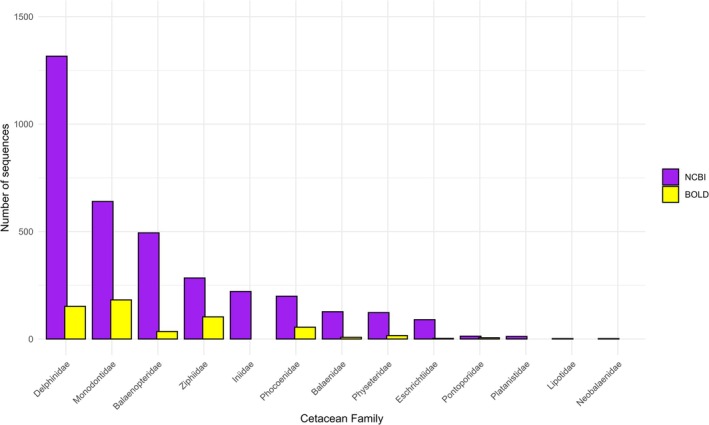
Number of COI and COI5‐P sequences available for each cetacean family in NCBI (purple bars) and BOLD (yellow bars).

The analysis of publicly available COI sequences for cetacean families revealed marked differences in representation between the NCBI Nucleotide and BOLD System databases (Figure 5). In NCBI, the most represented families are typically odontocetes, with Delphinidae (1316 sequences) leading, followed by Monodontidae (640), Balaenopteridae (494), and Ziphiidae (284). Other families showed lower coverage, with Lipotidae and Neobalaenidae represented by only two sequences each. In contrast, BOLD contained fewer sequences overall. The best‐represented families in BOLD are Monodontidae (182 sequences), Delphinidae (152), and Ziphiidae (103), while several families, including Iniidae, Platanistidae, Lipotidae, and Neobalaenidae, were absent from BOLD despite being present in NCBI. Even removing NCBI mined records, the vast majority of BOLD sequences also possess an associated NCBI accession number.

## 4 Discussion

This study evaluated the completeness, structure, and metadata quality of mitochondrial reference data for cetaceans in public genetic repositories to assess their suitability for biodiversity monitoring and eDNA applications. Cetaceans show comparatively strong taxonomic representation, with all recognised species present in at least one mitochondrial marker, suggesting current databases broadly support species detection. However, this apparent completeness masks structural imbalances, including uneven representation across families, loci, and geographic regions, as well as inconsistencies in taxonomy and metadata reporting. While these gaps do not necessarily prevent species identification, they can reduce confidence, comparability, and interpretability in downstream applications. Our findings therefore reveal a dual reality: cetacean reference datasets are relatively robust, yet their full utility depends on continued curation, metadata standardisation, and targeted sequencing to support reliable integration into molecular ecology frameworks.

### 4.1 Data Extraction, Marker Abundance, and Co‐Occurrence

The Delphinidae and Balaenopteridae cetacean families are predominant in the NCBI Nucleotide database with a comparatively high sequence representation, most likely due to their worldwide distribution (Pompa 2011) and high species diversity, which make them frequent targets of genetic research (Guo et al. 2022; Lo et al. 2025; McGowen 2011). Nevertheless, Delphinidae species are unevenly represented, with the top 10 species with the most unique accessions account for about 66.3% of all family sequences, whereas the 10 most underrepresented species contribute only 1.2% of the dataset. Notably, underrepresentation is not always genus‐wide, since genera such as Sousa and Cephalorhynchus include both underrepresented ( *S. sahulensis* , *S. teuszi*, *S. plumbea* , *C. cruciger* , *C. eutropia* and *C. hectori* ) and well‐represented species ( *S. chinensis* , *C. heavisidii* and *C. commersonii* ). This pattern indicates that genetic coverage reflects species‐specific constraints, including restricted or patchy geographic ranges, low‐density localised habitats with limited survey effort (e.g., *S. sahulensis* ; Jefferson and Rosenbaum 2014; Parra 2023), narrow endemic distributions with few documented populations (e.g., *S. teuszii* ; Weir and Collins 2015), and conservation or permitting frameworks that restrict tissue sampling (Weir et al. 2011). A similar pattern occurs in Balaenopteridae, where the four most represented species comprise 83.7% of all unique accessions. This is largely driven by the dominant presence of humpback whale (*Megaptera novaengliae*) sequences, a species that attracts disproportionate research attention due to its global distribution, high visibility, and historical and ongoing genetic monitoring programs (Baker et al. 1993; Jackson et al. 2014). Rice's whale (*Balaenoptera ricei*) is underrepresented in genetic databases, likely reflecting its recent recognition as a distinct species (Rosel et al. 2021). Because this lineage was historically grouped within the Bryde's whale ( *Balaenoptera edeni* ) complex, earlier sequences were deposited under prior taxonomy, and only a small number of accessions appear under the updated name, including four records dated 2012–2019 that reflect transitional classification. For eDNA applications, such taxonomic lag can propagate historical labels in reference databases, potentially limiting resolution under revised species frameworks.

Other families show similar patterns. Monodontidae, a monophyletic Arctic lineage comprising beluga whales ( *Delphinapterus leucas* ) and narwhals ( *Monodon monoceros* ), has received substantial sequencing attention (Lowry, Laidre, and Reeves 2017; Lowry, Reeves, and Laidre 2017). This is driven largely by belugas, whose predictable coastal aggregations and summer accessibility enable systematic sampling and genomic monitoring (Hornby et al. 2017). Recent work analysing more than 900 historical and contemporary samples from eastern Canada underscores sustained targeted sequencing (Montana et al. 2024). Narwhals, by contrast, occupy deeper offshore habitats and depend strongly on sea ice (Heide‐Jørgensen and Acquarone 2002; Skovrind et al. 2019), factors that likely contribute to their comparatively lower representation. Also, the Eschrichtiidae family, represented solely by the grey whale ( *Eschrichtius robustus* ), presents a relatively high number of sequences. Heavily exploited by whaling, grey whale populations in the North Pacific have more recently stabilised (Cooke 2018; Swartz 2018), which, along with ongoing conservation interest, may have contributed to increased sequencing effort. In contrast, the Ziphiidae family remains underrepresented. These deep‐diving, globally distributed species (MacLeod et al. 2005) are rarely encountered because they inhabit pelagic regions difficult to sample (Baird 2019; Tyack et al. 2006), and stranding events are comparatively uncommon (Dalebout et al. 2004), limiting opportunities for sequencing. Within this family, the Northern bottlenose whale (*Hyperodon ampullatus*) and the Goose beaked whale ( *Ziphius cavirostris* ) show a comparatively higher representation, while major genetic data gaps exist for the *Mesoplodon* and *Berardius* species. The Phocoenidae are well represented in the dataset, albeit with a lower number of records per species. Although distributed across coastal waters of the Pacific and Atlantic (Pompa 2011), their elusive nature and often small populations can complicate genetic sampling (Parsons et al. 2018). Nonetheless, recent global and regional scale genomic work has targeted harbour porpoise ( *Phocoena phocoena* ) populations (Ben Chehida et al. 2021, 2023) through strandings, improving data representation for this species. As expected, river dolphins remain underrepresented, given the small population sizes, limited distributions, and the consequently localised research efforts (Campbell et al. 2022). Lastly, Neobalaenidae, represented solely by the pygmy right whale ( *Caperea marginata* ), shows a marked lack of records, likely reflecting its remote Southern Hemisphere distribution, elusive small‐group behaviour, and limited regional research and sampling effort (Kim et al. 2025; Wolf et al. 2023). Identifying and understanding sequencing biases is essential to ensure prioritisation of underrepresented taxa such as the Ziphiidae, Neobalaenidae, and poorly sampled species within larger cetacean families, guiding targeted sampling campaigns, coordinated sequencing initiatives, and strategic data deposition efforts aimed at closing reference gaps and improving the reliability of downstream genetic and eDNA applications.

Unlike other taxonomic groups where taxonomic coverage is lower (Garg et al. 2019; van den Burg and Vieites 2023), all 103 cetacean species recognised by the NCBI Taxonomy are represented in the NCBI Nucleotide database. This confirms the broad species coverage introduced above, while reinforcing that uneven locus representation remains a practical limitation for detection confidence and comparability, particularly for less represented taxa. It is important to note that the number of valid species in the NCBI Taxonomy was used. This database tracks sequences present in NCBI (Federhen 2012), key for taxonomic matches of eDNA sequences. However, discrepancies exist between the NCBI Taxonomy and the infraorder classification accepted by the Society for Marine Mammalogy (SMM) or the World Register of Marine Species (WoRMS), which recognise 94 and 96 extant species, respectively (Society for Marine Mammalogy 2025; WoRMS 2025). For example, the Burrunan dolphin (*Tursiops australis*), proposed as a distinct species in 2011 (Charlton‐Robb et al. 2011), is included in the NCBI and accepted in WoRMS, but not considered by the SMM. Within the Iniidae family, the NCBI lists four species, whereas SMM recognises only one—the Amazon river dolphin ( *Inia geoffrensis* ). Unrecognised species include the Araguaian river dolphin ( *I. araguaiaensis* ), recently described as a new species but still lacking broad consensus (de Mello et al. 2025; Hrbek et al. 2014), and the Orinoco river dolphin ( *I. humboldtiana* ), often considered a subspecies (Boede et al. 2018). WoRMS currently recognises *I. araguaiaensis* and *I. boliviensis* as distinct species, aligning more closely with the NCBI treatment than with the SMM classification. Lastly, the Bolivian river dolphin, although presented as a species by the NCBI Taxonomy ( *I. boliviensis* ), is only recognised as an *I. geoffrensis* subspecies by the SMM. Bryde's whale ( *Balaenoptera edeni brydei* ) is treated as a subspecies by the SMM but as a distinct species in the NCBI, with ongoing research addressing this uncertainty (Lin et al. 2025). WoRMS similarly treats Bryde's whale within the broader *B. edeni* framework, reflecting continued taxonomic caution in this complex. A comparable case concerns the pygmy right whale ( *Caperea marginata* ), for which phylogenetic analyses have proposed reassignment to the resurrected family Cetotheriidae (Fordyce and Marx 2013), while major curated authorities such as WoRMS and the SMM continue to recognise Neobalaenidae. The NCBI also includes species with outdated nomenclature according to SMM, such as *Stenella plagiodon* (Perrin et al. 1987), or invalid genera like *Sagmatias*. WoRMS currently recognises *Sagmatias* as a valid genus, whereas the SMM retains these species within *Aethalodelphis* and *Cephalorhynchus*, illustrating how even curated authorities differ in their treatment of cetacean taxonomy. There is also a family‐level discrepancy where Kogiidae is not considered distinct from Physeteridae despite phylogenetic support for separation (McGowen et al. 2020). These inconsistencies arise because NCBI relies on submitters to provide taxonomic designations, and there is no automatic mechanism to update records when new taxonomic revisions are published. Updates potentially occur only when taxonomic revisions formally proposed in the peer‐reviewed literature, typically by taxonomists using genetic, morphological, or integrative evidence, are evaluated and incorporated by NCBI Taxonomy curators into the database (Schoch et al. 2020), or when submitters revise their entries in accordance with recognised taxonomic frameworks. This approach prioritises database continuity (Federhen 2012; Sayers et al. 2021; Schoch et al. 2020), so taxonomic revisions are integrated carefully to preserve identifier stability, which can delay the incorporation of updated classifications. Discrepancies between databases like the NCBI and authoritative references such as the SMM can challenge genomic and metabarcoding studies, potentially causing ambiguous sequence matches or outdated classifications that affect biodiversity assessments (Keck et al. 2023). Dedicated database curation strategies are therefore relevant to mitigate these issues and ensure alignment with current taxonomy (Crandall et al. 2023; Jeunen et al. 2023).

Sequence abundance patterns show that the mitochondrial control region (D‐loop) far exceeds other markers in the NCBI, with most records representing isolated sequences. This trend occurs across families, except Monodontidae, where markers show comparable abundance. The dominance of the D‐loop, also reflected in marker co‐occurrence, likely stems from both biological and methodological factors: its high polymorphism makes it the preferred marker for population genetics, generating numerous haplotypes per species and potentially introducing redundancy among deposited records. Biologically, the D‐loop exhibits the highest substitution rate within the mitochondrial genome because it is largely non‐coding and therefore under weaker functional constraints, allowing mutations to accumulate more freely (Hoelzel et al. 1991). This high variability makes it particularly informative for population‐level and phylogeographic studies in marine top predators (Antonacci et al. 2023; Gómez‐Lobo et al. 2024; Sigsgaard et al. 2020; Piboon et al. 2022), where fine‐scale resolution of maternal lineages is essential. Methodologically, the D‐loop is short and flanked by conserved tRNA genes, facilitating full marker sequencing (Cheng et al. 2012). D‐loop sequences have been consistently targeted across decades, underscoring their long‐standing role as the preferred mitochondrial marker. For these reasons, it became the standard marker in early cetacean population genetics, generating a large historical dataset that continues to be used today.

After D‐loop, most sequences presented the five markers, typical of complete mitochondrial genome sequencing. The number of complete mitochondrial genomes has steadily increased, being highest in 2021–2025, suggesting a gradual shift toward full mitochondrial genome approaches. This pattern likely reflects the broader transition toward genome‐scale sequencing in molecular ecology, where advances in high‐throughput technologies have made recovery of complete mitochondrial genomes increasingly routine, often as a by‐product of whole‐genome sequencing workflows. The rapid expansion of mitochondrial genome datasets across taxa mirrors this wider genomic trend, driven by declining sequencing costs and the integration of genomic methods into biodiversity research (Goodwin et al. 2016; Leigh et al. 2021; Smith 2016). Nevertheless, the prevalence of isolated sequences reflects practical considerations, as targeting a single gene is less expensive and labour‐intensive, allowing researchers to obtain sufficient data for population studies without the higher costs of whole‐genome approaches (Chen et al. 2021). As a result, single‐marker sequencing remains the dominant strategy for metabarcoding and genetic monitoring efforts in cetaceans. The Cytb and COI markers also show notable representation in NCBI. In contrast to the D‐loop, these protein‐coding regions are subject to stronger functional constraints, which limit their intraspecific variability but make them highly conserved across species (May‐Collado and Agnarsson 2006; Morón‐López et al. 2022). These markers are then suitable for species‐level identification and phylogenetic analyses, including DNA barcoding efforts (Hebert et al. 2003). Ribosomal genes (12S and 16S rRNA) are less represented in NCBI compared to other mitochondrial markers, despite being commonly used in recent eDNA metabarcoding studies of marine vertebrates (Miya et al. 2015; Ushio et al. 2025; Valsecchi et al. 2020; Wang et al. 2023). Like the other markers described above, these ribosomal genes evolve slowly due to strong functional constraints, making them relatively conserved within the otherwise variable mitochondrial genome (Yang et al. 2014). As such, they are ideal for universal eDNA approaches that aim to capture biodiversity across multiple taxa. However, their high conservation can limit species‐level resolution in some groups, representing a trade‐off between universality and the ability to discriminate closely related taxa (Afonso et al. 2024; Valsecchi et al. 2021). Although the comparatively lower number of ribosomal sequences is consistent with their primary role in species identification rather than population‐level studies, expanding sequencing efforts targeting these conserved markers remains an optimal strategy for strengthening eDNA reference datasets, particularly by improving taxonomic coverage and detection confidence.

Marker co‐occurrence across many accessions highlights the value of multi‐locus approaches, which enhance resolution, mitigate single‐gene limitations, and represent best practice for cetacean genetics by supporting analyses of population structure, phylogeography, biodiversity, and eDNA applications (Crowley et al. 2025; Sard et al. 2019; Weitemier et al. 2021). Realising this potential depends on addressing gaps in public databases, where partial marker representation underscores the need for more balanced sequencing and deposition of multiple mitochondrial genes.

### 4.2 Metadata Completeness and Geographic Coverage

Complete and accurate metadata are essential for effective molecular ecology applications. Geographic information contextualises sequences within ecological and biogeographical frameworks, supporting analyses of population structure, species distributions, local adaptation, regional primer design, and metabarcoding workflows that rely on curated local databases and reliable taxonomic assignment (Ma et al. 2016; Sigsgaard et al. 2016). Incomplete or ambiguous metadata can obscure biogeographic patterns, limiting the interpretability of genetic data at regional scales (Teixeira et al. 2025). These needs align with FAIR data principles (Findable, Accessible, Interoperable, Reusable), which emphasise comprehensive spatial metadata to improve discoverability and reuse (Wilkinson et al. 2016; Takahashi et al. 2025). However, cetacean genetic datasets show clear geographic metadata gaps consistent with broader assessments in other taxa (Peng et al. 2023). Although sampling locations can sometimes be inferred from associated publications, the absence of structured geographic metadata reduces findability for location‐based queries and limits reuse, reflecting deficiencies relative to FAIR principles that call for rich, searchable metadata (F2), sufficient contextual detail to support reuse (R1), and standardised vocabularies to enable interoperability (I1–I2) (Wilkinson et al. 2016).

The persistence of such gaps underscores the need for clearer submission standards and user‐friendly metadata templates at the time of sequence deposition. Addressing this issue is particularly important for cetaceans, as many species exhibit genetic differentiation between oceans (Jackson et al. 2014; LeDuc et al. 2007) and even within adjacent areas (Ben Chehida et al. 2023; Wiszniewski et al. 2010). Therefore, we encourage publication of complete metadata and recommend that original sequence submitters retrospectively update existing records to include missing information. Although the NCBI does not enforce retrospective submission, voluntary updates would greatly enhance the utility of genetic databases for population genetics, eDNA studies, and ultimately conservation and management.

Accounting for both the geographical distribution and coverage of the different families and genetic markers, the maps presented in this study represent only a minority of all the records included in the study, and therefore, an associated margin of error should be acknowledged. In terms of the number of unique accessions, sequencing efforts have been more intensive in the Northern Hemisphere and the equatorial Pacific, particularly across North America, Central America, and eastern Asia, as well as in the American sector of the Arctic Ocean and the western Atlantic. In addition to national differences in research funding and capacity, this uneven geographical representation likely reflects disparities in accessibility, research infrastructure, and historical sampling focus, as has been observed in terrestrial taxa (Šmíd 2022; Tydecks et al. 2018). Regions with well‐established monitoring programs, reliable logistics, and strong international collaborations have generated a disproportionate share of available data, whereas remote or less‐developed areas remain underrepresented due to limited infrastructure and challenging field conditions (Pitogo 2025). This pattern is also evident in the marine areas adjacent to parts of the African coastline, where previous studies have reported a lack of baseline information on cetacean occurrence (Braulik et al. 2018; Correia et al. 2024; Elwen et al. 2023), underscoring the need for scientific collaborations to support global equity in marine research, thereby benefiting conservation efforts worldwide.

Regarding the distribution of accessions by cetacean family, the geographic clustering by family is consistent with known biogeographical ranges, with several noteworthy exceptions. In Antarctica, for example, only the Balaenopteridae family is represented. Records from this region are expected, given the seasonal migrations to polar feeding grounds (Friedlaender et al. 2006). However, Antarctic waters host high cetacean diversity, including species from other families (Ainley et al. 2017; Pitman and Ensor 2003; Thiele et al. 2000). In European clusters, the Balaenopteridae is weakly represented despite its wide distribution and high abundance in the northeast Atlantic (Gilles et al. 2023), and the well‐documented fin whale ( *Balaenoptera physalus* ) population in the Mediterranean Sea (Aïssi et al. 2008; Castellote et al. 2012; Espada et al. 2024; Notarbartolo‐di‐Sciara et al. 2003; Panigada et al. 2024). This pattern likely reflects misrepresentation arising from incomplete metadata. Monodontidae predominance in Arctic clusters aligns with the sustained monitoring infrastructure described earlier, particularly long‐term programs run by NOAA and Fisheries and Oceans Canada (Fisheries and Oceans Canada 2022; NOAA Fisheries 2022; Parent et al. 2025).

Geographical clustering requires careful interpretation, especially when dealing with incomplete geographic metadata. For example, river dolphin families are associated with oceanic clusters that correspond closely to their respective freshwater habitats. In this dataset, Lipotidae records lack sufficient metadata for spatial assignment; however, their origin is clear, as the sole species, the baiji ( *Lipotes vexillifer* ), is endemic to the Yangtze River in China (Smith et al. 2017). Another example concerns harbour porpoise ( *Phocoena phocoena* ) sequences reported from Turkey, which likely originate from the resident Black Sea population, as the species is largely absent from the Mediterranean aside from rare incursions (Fontaine 2016; Pierce et al. 2024; Reeves and Notarbartolo di Sciara 2006). These examples illustrate how incomplete or imprecise geographic metadata can force researchers to infer sampling origins from species ecology rather than explicit annotation. Reliable reuse of public genetic data should not depend on such inference, underscoring the importance of standardised geographic metadata at the time of submission.

Given the previously discussed dominance and variability of the D‐loop, its global representation highlights this region as a candidate worth exploring for cetacean metabarcoding applications. For example, Yoshitake et al. (2023) developed universal D‐loop amplification primers for fish eDNA samples and demonstrated that the region's variability enables species‐level resolution in biodiversity assessments, while also providing information on population size and structure, unlocking the potential of eDNA as a tool for resource estimation. Furthermore, successful species‐specific D‐loop primers have already been applied in marine predator eDNA studies (e.g., Parsons et al. 2025; Sigsgaard et al. 2016), indicating that expansion toward broader primer systems is not without precedent. Nevertheless, the D‐loop is challenging to target in universal eDNA approaches, as it exhibits substantial sequence heterogeneity among species, making it difficult to find conserved priming sites across taxa without species‐specific dropout (Yoshitake et al. 2023). Hence, considering long‐read sequencing via increasingly developed Nanopore or PacBio technologies (Chang et al. 2024; Doorenspleet et al. 2025) could be an interesting alternative, as these platforms can sequence longer mitochondrial fragments and potentially capture the full D‐loop region with high resolution. Rapid advances in long‐read error correction and vertebrate eDNA applications demonstrate improving feasibility for recovering informative mitochondrial regions beyond traditional short amplicons, even if current implementations remain technically demanding. However, such approaches require deep sequencing coverage to offset potential co‐amplification of other vertebrate DNA and remain relatively costly and untested at large scales. The effectiveness of any metabarcoding primer set also depends on the availability of reference sequences in the target region. Developing primers for markers such as COI or 16S in areas where local reference sequences are scarce can result in poor amplification and ambiguous species assignments. We provide insights into the distribution of genetic data across cetacean families, species, and mitochondrial markers, which may guide and inform future research efforts.

### 4.3 Database Comparison (NCBI vs. BOLD Systems)

In this article, we also analysed BOLD Systems records. This public repository, focused primarily on the COI gene, is widely used in studies targeting a broad range of taxa (Chen et al. 2025; Leduc et al. 2019; Moutinho et al. 2024). However, to our knowledge, no eDNA studies targeting cetaceans have used BOLD as the primary reference dataset. We therefore assessed its current status and potential usefulness for cetacean species identification. Our results indicate substantially lower coverage on this platform compared to NCBI. Although BOLD primarily curates its own submissions, it also integrates publicly available NCBI sequences to expand taxonomic coverage (Ratnasingham and Hebert 2007). This integration helps reduce barcode gaps and improve assignment accuracy, but many records may still be redundant across repositories. Recent eDNA metabarcoding studies show that multi‐reference database approaches enhance species detection and minimize misassignments, particularly where marker representation is uneven or incomplete (Arranz et al. 2020; Leray and Knowlton 2015; Meiklejohn et al. 2019). Strengthening interoperability between databases and promoting validated cetacean sequences is therefore crucial for improving the reliability and taxonomic resolution of future eDNA studies.

## 5 Conclusions

Reliable genetic reference databases form a critical foundation for modern biodiversity monitoring as eDNA approaches become increasingly central to marine research. Our analysis shows that mitochondrial reference resources for cetaceans are broadly capable of supporting species detection, yet their apparent completeness conceals structural imbalances that influence confidence, comparability, and long‐term utility. Although all recognised species are represented, uneven data distribution across families and loci reflects ecological accessibility, historical research focus, and taxonomic lag, identifying clear priorities for targeted sequencing. Marker patterns, particularly the dominance of the mitochondrial control region, illustrate how historical sequencing practices continue to shape present‐day reference utility, emphasising the need to align primer development with available data. At the same time, incomplete geographic metadata and inconsistencies among taxonomic authorities constrain interoperability, reproducibility, and regional interpretation, reinforcing the importance of standardised reporting, active curation, and retrospective metadata improvement. Addressing these interconnected gaps through coordinated sequencing efforts, improved metadata practices, and cross‐database harmonisation will strengthen cetacean reference frameworks and support more reliable, scalable eDNA applications for biodiversity monitoring, conservation, and management in an evolving molecular ecology landscape.

## Author Contributions

L.A., P.S.‐B., M.R.J., D.K.M., G.J.P., C.M., A.M.C.: conceptualisations. L.A., A.S.L.: methodology. A.S.L.: software. L.A.: validation. L.A., A.L., A.M.C.: formal analysis. L.A., A.L.: investigation. I.S.‐P., C.M., A.M.C.: resources. L.A., A.L.: data curation. L.A., A.S.L., A.L., P.S.‐B., M.A.‐G., R.V., M.R.J., C.S., D.K.M., I.S.‐P, G.J.P., C.M., A.M.C.: writing – original draft. L.A., A.L., A.S.L., R.V., M.R.J., A.M.C.: writing – review and editing. L.A., A.L., A.M.C.: visualisation. R.V., P.S.‐B., G.J.P., C.M., A.M.C.: supervision. A.M.C.: project administration. D.K.M., I.S.‐P, G.J.P., C.M., A.M.C.: funding acquisition.

## Funding

This research was funded by Biodiversa+, the European Biodiversity Partnership, in the context of the EMPHATIC Project under the 2022–2023 BiodivMon joint call. It was co‐funded by the European Commission (GA No. 101052342) and the following funding organisations: Fundación Biodiversidad (FB, Spain), Fundação para a Ciência e Tecnologia (FCT, Portugal), Agence Nationale de la Recherche (ANR, France), and the Ministero dell'Università e della Ricerca (MUR, Italy), and by national funds through FCT—Fundação para a Ciência e a Tecnologia, I.P., and by the European Commission's Recovery and Resilience Facility, within the scope of UID/04423/2025 (https://doi.org/10.54499/UID/04423/2025), UID/PRR/04423/2025 (https://doi.org/10.54499/UID/PRR/04423/2025), and LA/P/0101/2020 (https://doi.org/10.54499/LA/P/0101/2020). This work is also a result of the project ATLANTIDA II (ref. NORTE2030‐FEDER‐01799200), co‐financed by the European Union through the NORTE 2030 Regional Program and the European Regional Development Fund (ERDF). LA was supported by a PhD FCT grant (2024.04444.BD). This work was also conducted in the scope of MARCO‐BOLO and REDUCE projects, both funded by the European Union under the Horizon Europe Programme, Grant Agreement No. 101082021 and 101135583, respectively. PS was supported by the Spanish Ministry for the Ecological Transition and the Demographic Challenge (MITECO), through the Commission [28‐5307] to the IEO‐CSIC for the “Technical Scientific Advice for the Protection of the Marine Environment: Assessment and Monitoring of Marine Strategies, Monitoring of Marine Protected Areas of State Competence" (2018‐2021).

## Conflicts of Interest

The authors declare no conflicts of interest.

## Supporting information


**Figure S1:** (A, B, C, D). Marker abundance across cetacean species.
**Figure S2:** Geographic distribution of cumulative sequences across cetacean families.

**Table S1:** Full analysed dataset extracted from NCBI Nucleotide database (4th of July 2025) with accession numbers, presence or absence (TRUE/FALSE) of each target markers, journal, taxa information and geographical metadata extracted from the records. Cluster locations and coordinates, attributed based on worldmap position of the records, were imported into ArcGIS for downstream analysis. Geo_loc_source additionally refers to where geographic information was derived from (original, filled with coordinates, or missing).
**Table S2:** Number of total and target marker (12S, 16S, COI, Cytb and D‐loop) sequences at species level, from the extracted dataset from the NCBI Nucleotide database (accessed on July 4, 2025). NCBI taxonomy correspond to species/subspecies associated to at least one unique accession, while SMM Taxonomy indicates its currently accepted taxonomic classification (Society of Marine Mammalogy, 2025).
**Table S3:** Full analysed COI/COI‐5P dataset extracted from Barcode of Life Database Systems (BOLD; 4th of July 2025) with processid, sampleid, recordID, bin, taxa information, geographical metadata and GenBank related information.
**Table S4:** Number of records per cetacean family in each of the formed geographical clusters, extracted from NCBI Nucleotide database (4th of July 2025). Only records with geographical information available.
**Table S5:** Number of studies and marker sequences for each of the formed geographical clusters, of sequences extracted from NCBI Nucleotide database (4th of July 2025). Only records with geographical information available.

## Data Availability

All sequence data analysed in this study were obtained from the publicly accessible NCBI Nucleotide database. The complete curated dataset used for analysis, including accession numbers, is provided in the Supporting Information and has been archived in Zenodo (https://zenodo.org/records/17733928). No new data were generated in this study.

## References

[men70141-bib-0001] Afonso, L. , J. Costa , A. M. Correia , et al. 2024. “Environmental DNA as a Complementary Tool for Biodiversity Monitoring: A Multi‐Technique and Multi‐Trophic Approach to Investigate Cetacean Distribution and Feeding Ecology.” PLoS One 19, no. 10: e0300992. 10.1371/journal.pone.0300992. 39413078 PMC11482729

[men70141-bib-0002] Ainley, D. G. , K. Lindke , G. Ballard , et al. 2017. “Spatio‐Temporal Occurrence Patterns of Cetaceans Near Ross Island, Antarctica, 2002–2015: Implications for Food Web Dynamics.” Polar Biology 40, no. 9: 1761–1775. 10.1007/s00300-017-2100-9.

[men70141-bib-0003] Aïssi, M. , A. Celona , G. Comparetto , R. Mangano , M. Würtz , and A. Moulins . 2008. “Large‐Scale Seasonal Distribution of Fin Whales (Balaenoptera physalus) in the Central Mediterranean Sea.” Journal of the Marine Biological Association of the United Kingdom 88, no. 6: 1253–1261. 10.1017/S0025315408000891.

[men70141-bib-0140] Alexandre, B. G. , M. M. Cruz , K. B. do Amaral , L. S. Hoffmann , T. R. O. de Freitas , and R. Zanini . 2024. “Exploring mtDNA Databases to Evaluate the Population Structure and Genetic Diversity of *Tursiops truncatus* in the Atlantic Ocean: Implications for the Conservation of a Small, Offshore Population.” Ecologies 5, no. 2: 170–187. 10.3390/ecologies5020011.

[men70141-bib-0004] Allaire, J. J. , P. Ellis , C. Gandrud , et al. 2025. “networkD3: D3 JavaScript Network Graphs from R (Version 0.4.1). Comprehensive R Archive Network.” 10.32614/CRAN.package.networkD3.

[men70141-bib-0005] Antonacci, R. , G. Linguiti , F. Paradiso , et al. 2023. “Mitochondrial DNA Diversity and Genetic Structure of Striped Dolphin Stenella coeruleoalba in the Northern Ionian Sea.” Frontiers in Marine Science 10: 1088598. 10.3389/fmars.2023.1088598.

[men70141-bib-0006] Arranz, V. , W. S. Pearman , J. D. Aguirre , and L. Liggins . 2020. “MARES, a Replicable Pipeline and Curated Reference Database for Marine Eukaryote Metabarcoding.” Scientific Data 7, no. 1: 209. 10.1038/s41597-020-0549-9. 32620910 PMC7334202

[men70141-bib-0007] Baird, R. W. 2019. “Behavior and Ecology of Not‐So‐Social Odontocetes: Cuvier's and Blainville's Beaked Whales.” In Ethology and Behavioral Ecology of Odontocetes, 305–329. Springer International Publishing. 10.1007/978-3-030-16663-2_14.

[men70141-bib-0008] Baker, C. S. , A. Perry , J. L. Bannister , et al. 1993. “Abundant Mitochondrial DNA Variation and World‐Wide Population Structure in Humpback Whales.” Proceedings of the National Academy of Sciences 90, no. 17: 8239–8243. 10.1073/pnas.90.17.8239. PMC47324 8367488

[men70141-bib-0009] Baker, C. S. , D. Steel , S. Nieukirk , and H. Klinck . 2018. “Environmental DNA (eDNA) From the Wake of the Whales: Droplet Digital PCR for Detection and Species Identification.” Frontiers in Marine Science 5: 133. 10.3389/fmars.2018.00133.

[men70141-bib-0010] Ben Chehida, Y. , R. Loughnane , J. Thumloup , et al. 2021. “No Leading‐Edge Effect in North Atlantic Harbor Porpoises: Evolutionary and Conservation Implications.” Evolutionary Applications 14, no. 6: 1588–1611. 10.1111/eva.13227. 34178106 PMC8210799

[men70141-bib-0011] Ben Chehida, Y. , T. Stelwagen , J. P. Hoekendijk , et al. 2023. “Harbor Porpoise Losing Its Edge: Genetic Time Series Suggests a Rapid Population Decline in Iberian Waters Over the Last 30 Years.” Ecology and Evolution 13, no. 12: e10819. 10.1002/ece3.10819. 38089896 PMC10714065

[men70141-bib-0012] Blackman, R. C. , J.‐C. Walser , L. Rüber , et al. 2023. “General Principles for Assignments of Communities From eDNA: Open Versus Closed Taxonomic Databases.” Environmental DNA 5: 326–342. 10.1002/edn3.382.

[men70141-bib-0013] Boede, E. O. , E. Mujica‐Jorquera , F. Boede , and C. Varela . 2018. “Reproductive Management of the Orinoco River Dolphin Inia geoffrensis humboldtiana in Venezuela.” International Zoo Yearbook 52, no. 1: 245–257. 10.1111/izy.12195.

[men70141-bib-0014] Braulik, G. T. , M. Kasuga , A. Wittich , et al. 2018. “Cetacean Rapid Assessment: An Approach to Fill Knowledge Gaps and Target Conservation Across Large Data Deficient Areas.” Aquatic Conservation: Marine and Freshwater Ecosystems 28, no. 1: 216–230. 10.1002/aqc.2833.

[men70141-bib-0015] Campbell, E. , J. Alfaro‐Shigueto , E. Aliaga‐Rossel , et al. 2022. “Challenges and Priorities for River Cetacean Conservation.” Endangered Species Research 49: 13–42. 10.3354/esr01201.

[men70141-bib-0016] Castellote, M. , C. W. Clark , and M. O. Lammers . 2012. “Fin Whale (Balaenoptera physalus) Population Identity in the Western Mediterranean Sea.” Marine Mammal Science 28, no. 2: 325–344. 10.1111/j.1748-7692.2011.00491.x.

[men70141-bib-0017] Chang, J. J. M. , Y. C. A. Ip , W. L. Neo , M. A. Mowe , Z. Jaafar , and D. Huang . 2024. “Primed and Ready: Nanopore Metabarcoding Can Now Recover Highly Accurate Consensus Barcodes That Are Generally Indel‐Free.” BMC Genomics 25, no. 1: 842. 10.1186/s12864-024-10767-4. 39251911 PMC11382387

[men70141-bib-0018] Charlton‐Robb, K. , L.‐A. Gershwin , R. Thompson , J. Austin , K. Owen , and S. McKechnie . 2011. “A New Dolphin Species, the Burrunan Dolphin (*Tursiops australis sp. Nov*.), endemic to Southern Australian Coastal Waters.” PLoS One 6, no. 9: e24047. 10.1371/journal.pone.0024047. 21935372 PMC3173360

[men70141-bib-0019] Chen, R. , M. A. Aldred , W. Xu , J. Zein , P. Bazeley , and S. A. Comhair . 2021. “Comparison of Whole Genome Sequencing and Targeted Sequencing for Mitochondrial DNA.” Mitochondrion 58: 303–310. 10.1016/j.mito.2021.01.006. 33513442 PMC8354572

[men70141-bib-0020] Chen, X. , Z. Yan , S. Li , and M. Yao . 2025. “Advancing Aquatic Biodiversity Assessments of Invertebrates Using eDNA Metabarcoding: A Systematic Evaluation of Primers for Marine and Freshwater Communities.” Methods in Ecology and Evolution 16: 2408–2430. 10.1111/2041-210X.70152.

[men70141-bib-0021] Cheng, Y. Z. , T. J. Xu , X. X. Jin , et al. 2012. “Universal Primers for Amplification of the Complete Mitochondrial Control Region in Marine Fish Species.” Molecular Biology 46, no. 5: 727–730. 10.1134/S0026893312040024. 23156681

[men70141-bib-0022] Claver, C. , O. Canals , L. G. de Amézaga , I. Mendibil , and N. Rodriguez‐Ezpeleta . 2023. “An Automated Workflow to Assess Completeness and Curate GenBank for Environmental DNA Metabarcoding: The Marine Fish Assemblage as Case Study.” Environmental DNA 5, no. 4: 634–647. 10.1002/edn3.433.

[men70141-bib-0023] Cooke, J. G. 2018. “Eschrichtius robustus. The IUCN Red List of Threatened Species 2018: e.T8097A50353881.” 10.2305/IUCN.UK.2018-2.RLTS.T8097A50353881.en.

[men70141-bib-0024] Correia, A. M. , M. Mihova , Á. Gil , et al. 2024. “Cetaceans of North‐Western Continental Africa (Morocco to Liberia): Diversity and Distribution.” Frontiers in Marine Science 11: 1427334. 10.3389/fmars.2024.1427334.

[men70141-bib-0025] Crandall, E. D. , R. H. Toczydlowski , L. Liggins , et al. 2023. “Importance of Timely Metadata Curation to the Global Surveillance of Genetic Diversity.” Conservation Biology 37, no. 4: e14061. 10.1111/cobi.14061. 36704891 PMC10751740

[men70141-bib-0026] Crowley, S. E. , T. Kess , P. Bentzen , V. K. Neville , C. Bloom , and N. Smith . 2025. “eDNA Metabarcoding and Whole Genome Sequencing Detect European–American Eel Hybrids in Northeastern Canada.” Canadian Journal of Fisheries and Aquatic Sciences 82: 1–11. 10.1139/cjfas-2024-0371.

[men70141-bib-0027] Dalebout, M. L. , C. S. Baker , J. G. Mead , V. G. Cockcroft , and T. K. Yamada . 2004. “A Comprehensive and Validated Molecular Taxonomy of Beaked Whales, Family Ziphiidae.” Journal of Heredity 95, no. 6: 459–473. 15475391 10.1093/jhered/esh054

[men70141-bib-0028] de Mello, D. M. , W. Gravena , A. Duarte‐Benvenuto , A. S. Lima , F. R. Gomes , and V. M. da Silva . 2025. “Comprehensive Assessment of the Physical and Health Features of the Threatened Araguaian River Dolphin Inia araguaiaensis .” PLoS One 20, no. 3: e0319212. 10.1371/journal.pone.0319212. 40163480 PMC11957337

[men70141-bib-0029] Doorenspleet, K. , L. Jansen , S. Oosterbroek , et al. 2025. “The Long and the Short of It: Nanopore‐Based eDNA Metabarcoding of Marine Vertebrates Works; Sensitivity and Species‐Level Assignment Depend on Amplicon Lengths.” Molecular Ecology Resources 25, no. 4: e14079. 10.1111/1755-0998.14079. 39930907 PMC11969631

[men70141-bib-0030] Dubois, S. , and S. Chamberlain . 2025. “bold: Interface to Bold Systems API (R package version 1.3.0) [Computer software].” https://github.com/ropensci/bold.

[men70141-bib-0031] Elwen, S. , J. Fearey , E. Ross‐Marsh , et al. 2023. “Cetacean Diversity of the Eastern South Atlantic Ocean and Vema Seamount Detected During a Visual and Passive Acoustic Survey, 2019.” Journal of the Marine Biological Association of the United Kingdom 103: e41. 10.1017/S0025315423000255.

[men70141-bib-0032] Espada, R. , A. Camacho‐Sánchez , L. Olaya‐Ponzone , E. Martín‐Moreno , D. Patón , and J. C. García‐Gómez . 2024. “Fin Whale Balaenoptera physalus Historical Sightings and Strandings, Ship Strikes, Breeding Areas and Other Threats in the Mediterranean Sea: A Review (1624–2023).” Environments 11, no. 6: 104. 10.3390/environments11060104.

[men70141-bib-0033] Evans, P. G. , and P. S. Hammond . 2004. “Monitoring Cetaceans in European Waters.” Mammal Review 34, no. 1–2: 131–156.

[men70141-bib-0035] Federhen, S. 2012. “The NCBI Taxonomy Database.” Nucleic Acids Research 40, no. D1: D136–D143. 10.1093/nar/gkr1178. 22139910 PMC3245000

[men70141-bib-0036] Fisheries and Oceans Canada . 2022. “Report on the Progress of Recovery Strategy Implementation for the Beluga Whale (Delphinapterus leucas) (DFO), St. Lawrence Estuary Population in Canada, for the Period 2012 to 2019. Species at Risk Act Recovery Strategy Report Series.”

[men70141-bib-0037] Fleming, J. F. 2023. “The Wealth of Shared Resources: Improving Molecular Taxonomy Using eDNA and Public Databases.” Zoologica Scripta 52, no. 3: 226–234. 10.1111/zsc.12591.

[men70141-bib-0038] Fontaine, M. C. 2016. “Harbour Porpoises, Phocoena phocoena, in the Mediterranean Sea and Adjacent Regions: Biogeographic Relicts of the Last Glacial Period.” Advances in Marine Biology 75: 333–358. 10.1016/bs.amb.2016.08.006. 27770989

[men70141-bib-0039] Fordyce, R. E. , and F. G. Marx . 2013. “The Pygmy Right Whale Caperea marginata: The Last of the Cetotheres.” Proceedings of the Royal Society B: Biological Sciences 280, no. 1753: 20122645. 10.1098/rspb.2012.2645. PMC3574355 23256199

[men70141-bib-0040] Friedlaender, A. S. , P. N. Halpin , S. S. Qian , et al. 2006. “Whale Distribution in Relation to Prey Abundance and Oceanographic Processes in Shelf Waters of the Western Antarctic Peninsula.” Marine Ecology Progress Series 317: 297–310. 10.3354/meps317297.

[men70141-bib-0041] Garg, A. , D. Leipe , and P. Uetz . 2019. “The Disconnect Between DNA and Species Names: Lessons From Reptile Species in the NCBI Taxonomy Database.” Zootaxa 4706, no. 3: 401–407. 10.11646/zootaxa.4706.3.1. 32230528

[men70141-bib-0042] Gilles, A. , M. Authier , N. C. Ramirez‐Martinez , H. Araújo , A. Blanchard , and J. Carlström . 2023. “Estimates of cetacean abundance in European Atlantic waters in summer 2022 from the SCANS‐IV aerial and shipboard surveys. Final report published 29 September 2023. 64.”

[men70141-bib-0043] Gold, Z. , A. R. Wall , T. M. Schweizer , et al. 2022. “A Manager's Guide to Using eDNA Metabarcoding in Marine Ecosystems.” PeerJ 10: e14071. 10.7717/peerj.14071. 36405018 PMC9673773

[men70141-bib-0044] Gómez‐Lobo, D. A. , A. P. Monteoliva , A. Fernandez , et al. 2024. “Mitochondrial Variation of Bottlenose Dolphins (Tursiops truncatus) From the Canary Islands Suggests a Key Population for Conservation With High Connectivity Within the North‐East Atlantic Ocean.” Animals 14, no. 6: 901. 10.3390/ani14060901. 38539998 PMC10967437

[men70141-bib-0045] Goodwin, S. , J. D. McPherson , and W. R. McCombie . 2016. “Coming of Age: Ten Years of Next‐Generation Sequencing Technologies.” Nature Reviews Genetics 17: 333–351. 10.1038/nrg.2016.49. PMC10373632 27184599

[men70141-bib-0046] Guo, W. , D. Sun , Y. Cao , et al. 2022. “Extensive Interspecific Gene Flow Shaped Complex Evolutionary History and Underestimated Species Diversity in Rapidly Radiated Dolphins.” Journal of Mammalian Evolution 29, no. 2: 353–367. 10.1007/s10914-021-09581-6.

[men70141-bib-0047] Hebert, P. D. , A. Cywinska , S. L. Ball , and J. R. DeWaard . 2003. “Biological Identifications Through DNA Barcodes.” Proceedings of the Royal Society of London. Series B: Biological Sciences 270, no. 1512: 313–321. 10.1098/rspb.2002.2218. PMC1691236 12614582

[men70141-bib-0048] Heide‐Jørgensen, M. P. , and M. Acquarone . 2002. “Size and Trends of the Bowhead Whale, Beluga Andnarwhal Stocks Wintering Off West Greenland.” NAMMCO Scientific Publications 4: 191–210.

[men70141-bib-0049] Hoelzel, A. R. , J. M. Hancock , and G. A. Dover . 1991. “Evolution of the Cetacean Mitochondrial D‐Loop Region.” Molecular Biology and Evolution 8, no. 4: 475–493. 10.1093/oxfordjournals.molbev.a040662. 1717809

[men70141-bib-0050] Hornby, C. A. , J. Iacozza , C. Hoover , D. G. Barber , and L. L. Loseto . 2017. “Beluga Whale Delphinapterus leucas Late Summer Habitat Use and Support for Foraging Areas in the Canadian Beaufort Sea.” Marine Ecology Progress Series 574: 243–257. 10.3354/meps12178.

[men70141-bib-0051] Hrbek, T. , V. M. F. da Silva , N. Dutra , W. Gravena , A. R. Martin , and I. P. Farias . 2014. “A New Species of River Dolphin From Brazil or: How Little Do We Know Our Biodiversity.” PLoS One 9, no. 1: e83623. 10.1371/journal.pone.0083623. 24465386 PMC3898917

[men70141-bib-0052] Jackson, J. A. , D. J. Steel , P. Beerli , et al. 2014. “Global Diversity and Oceanic Divergence of Humpback Whales (Megaptera novaeangliae).” Proceedings of the Royal Society B: Biological Sciences 281, no. 1786: 20133222. 10.1098/rspb.2013.3222. PMC4046397 24850919

[men70141-bib-0053] Jefferson, T. A. , and H. C. Rosenbaum . 2014. “Taxonomic Revision of the Humpback Dolphins (*Sousa spp*.), and Description of a New Species From Australia.” Marine Mammal Science 30, no. 4: 1494–1541. 10.1111/mms.12152.

[men70141-bib-0054] Jeunen, G. J. , E. Dowle , J. Edgecombe , U. von Ammon , N. J. Gemmell , and H. Cross . 2023. “Crabs—A Software Program to Generate Curated Reference Databases for Metabarcoding Sequencing Data.” Molecular Ecology Resources 23, no. 3: 725–738. 10.1111/1755-0998.13741. 36437603

[men70141-bib-0055] Keck, F. , M. Couton , and F. Altermatt . 2023. “Navigating the Seven Challenges of Taxonomic Reference Databases in Metabarcoding Analyses.” Molecular Ecology Resources 23, no. 4: 742–755. 10.1111/1755-0998.13746. 36478393

[men70141-bib-0056] Kim, Y. , T. Katsumata , T. Isoda , and K. Matsuoka . 2025. “Rare Sightings of the Pygmy Right Whale (Caperea marginata) During the 2022/2023 JASS‐A Cruise in the Southwestern Pacific.” Cetacean Population Studies 5: 7–18. 10.34331/cpops.2023-F-001.

[men70141-bib-0057] Kiszka, J. , M. Heithaus , and A. Wirsing . 2015. “Behavioural Drivers of the Ecological Roles and Importance of Marine Mammals.” Marine Ecology Progress Series 523: 267–281. 10.3354/meps11180.

[men70141-bib-0058] Kiszka, J. J. , M. S. Woodstock , and M. R. Heithaus . 2022. “Functional Roles and Ecological Importance of Small Cetaceans in Aquatic Ecosystems.” Frontiers in Marine Science 9: 803173. 10.3389/fmars.2022.803173.

[men70141-bib-0059] Krassowski, M. 2020. “krassowski/complex‐upset [Computer software]. Zenodo.” 10.5281/zenodo.3700590.

[men70141-bib-0060] Leduc, N. , A. Lacoursière‐Roussel , K. L. Howland , et al. 2019. “Comparing eDNA Metabarcoding and Species Collection for Documenting Arctic Metazoan Biodiversity.” Environmental DNA 1, no. 4: 342–358. 10.1002/edn3.35.

[men70141-bib-0061] LeDuc, R. G. , A. E. Dizon , M. Goto , et al. 2007. “Patterns of Genetic Variation in Southern Hemisphere Blue Whales and the Use of Assignment Test to Detect Mixing on the Feeding Grounds.” Journal of Cetacean Research and Management 9, no. 1: 73–80. 10.47536/jcrm.v9i1.694.

[men70141-bib-0062] Lee, K. , J. Lee , Y. Cho , et al. 2019. “First Report of the Complete Mitochondrial Genome and Phylogenetic Analysis of Fraser's Dolphin (Lagenodelphis hosei).” Conservation Genetics Resources 11: 47–50. 10.1007/s12686-017-0964-1.

[men70141-bib-0063] Leigh, D. M. , C. B. van Rees , K. L. Millette , et al. 2021. “Opportunities and Challenges of Macrogenetic Studies.” Nature Reviews Genetics 22: 791–807. 10.1038/s41576-021-00394-0. 34408318

[men70141-bib-0064] Leray, M. , and N. Knowlton . 2015. “DNA Barcoding and Metabarcoding of Standardized Samples Reveal Patterns of Marine Benthic Diversity.” Proceedings of the National Academy of Sciences 112, no. 7: 2076–2081. 10.1073/pnas.1424997112. PMC4343139 25646458

[men70141-bib-0065] Lin, Y. T. , F. Hui , W. Han , et al. 2025. “Chromosome‐Level Genome Assembly of Eden's Whale Clarifies the Taxonomy and Speciation of Bryde's Whale Complex.” Molecular Biology and Evolution 42, no. 10: msaf234. 10.1093/molbev/msaf234. 40973465 PMC12492004

[men70141-bib-0066] Lo, B. W. , F. Martinez Real , A. Magg , J. P. Wise Sr. , S. Mundlos , and P. Franchini . 2025. “Genome‐Wide Demographic Analyses of Balaenid Whales Revealed Complex History of Gene Flow Associated With Past Climate Oscillation.” Genome Biology and Evolution 17, no. 5: evaf081. 10.1093/gbe/evaf081. 40323022 PMC12082451

[men70141-bib-0067] López‐Oceja, A. , X. Lekube , L. Ruiz , J. A. Mujika‐Alustiza , and M. M. De Pancorbo . 2019. “CYT B L15601 and H15748 Primers for Genetic Identification of Cetacean Species.” Forensic Science International: Genetics Supplement Series 7, no. 1: 771–772. 10.1016/j.fsigss.2019.10.171.

[men70141-bib-0068] Lowry, L. , K. Laidre , and R. Reeves . 2017. “Monodon monoceros. The IUCN Red List of Threatened Species 2017: e.T13704A50367651.” 10.2305/IUCN.UK.2017-3.RLTS.T13704A50367651.en.

[men70141-bib-0069] Lowry, L. , R. Reeves , and K. Laidre . 2017. “*Delphinapterus leucas*. The IUCN Red List of Threatened Species 2017: E.T6335A50352346.” 10.2305/IUCN.UK.2017-3.RLTS.T6335A50352346.en.

[men70141-bib-0070] Ma, H. , K. Stewart , S. Lougheed , J. Zheng , Y. Wang , and J. Zhao . 2016. “Characterization, Optimization, and Validation of Environmental DNA (eDNA) Markers to Detect an Endangered Aquatic Mammal.” Conservation Genetics Resources 8: 561–568. 10.1007/s12686-016-0597-9.

[men70141-bib-0071] MacLeod, C. D. , W. F. Perrin , R. Pitman , et al. 2005. “Known and Inferred Distributions of Beaked Whale Species (*Cetacea*: Ziphiidae).” Journal of Cetacean Research and Management 7, no. 3: 271.

[men70141-bib-0072] Massicotte, P. , and A. South . 2025. “rnaturalearth: World Map Data from Natural Earth [R package].” https://docs.ropensci.org/rnaturalearth/.

[men70141-bib-0073] May‐Collado, L. , and I. Agnarsson . 2006. “Cytochrome b and Bayesian Inference of Whale Phylogeny.” Molecular Phylogenetics and Evolution 38, no. 2: 344–354. 10.1016/j.ympev.2005.09.019. 16325433

[men70141-bib-0074] McGowen, M. R. 2011. “Toward the Resolution of an Explosive Radiation—A Multilocus Phylogeny of Oceanic Dolphins (Delphinidae).” Molecular Phylogenetics and Evolution 60, no. 3: 345–357. 10.1016/j.ympev.2011.05.003. 21600295

[men70141-bib-0075] McGowen, M. R. , G. Tsagkogeorga , S. Álvarez‐Carretero , et al. 2020. “Phylogenomic Resolution of the Cetacean Tree of Life Using Target Sequence Capture.” Systematic Biology 69, no. 3: 479–501. 31633766 10.1093/sysbio/syz068 PMC7164366

[men70141-bib-0076] Meiklejohn, K. A. , N. Damaso , and J. M. Robertson . 2019. “Assessment of BOLD and GenBank–Their Accuracy and Reliability for the Identification of Biological Materials.” PLoS One 14, no. 6: e0217084. 10.1371/journal.pone.0217084. 31216285 PMC6584008

[men70141-bib-0077] Miya, M. , Y. Sato , T. Fukunaga , et al. 2015. “MiFish, a Set of Universal PCR Primers for Metabarcoding Environmental DNA From Fishes: Detection of More Than 230 Subtropical Marine Species.” Royal Society Open Science 2, no. 7: 150088. 10.1098/rsos.150088. 26587265 PMC4632578

[men70141-bib-0078] Montana, L. , T. T. Bringloe , A. Bourret , et al. 2024. “Reduced Representation and Whole‐Genome Sequencing Approaches Highlight Beluga Whale Populations Associated to Eastern Canada Summer Aggregations.” Evolutionary Applications 17, no. 12: 1–20. 10.1111/eva.70058. PMC11655672 39703673

[men70141-bib-0079] Morón‐López, J. , K. Vergara , M. Sato , G. Gajardo , and S. Ueki . 2022. “Intraspecies Variation of the Mitochondrial Genome: An Evaluation for Phylogenetic Approaches Based on the Conventional Choices of Genes and Segments on Mitochondrial Genome.” PLoS One 17, no. 8: e0273330. 10.1371/journal.pone.0273330. 35980990 PMC9387813

[men70141-bib-0080] Moutinho, J. , F. O. Costa , and S. Duarte . 2024. “Advancements in DNA Metabarcoding Protocols for Monitoring Zooplankton in Marine and Brackish Environments.” Journal of Marine Science and Engineering 12, no. 11: 2093. 10.3390/jmse12112093.

[men70141-bib-0081] NOAA Fisheries . 2022, November 2. “1979–2021 Aerial Surveys of Arctic Marine Mammals Historical Database. Alaska Fisheries Science Center.” https://www.fisheries.noaa.gov/resource/data/1979‐2021‐aerial‐surveys‐arctic‐marine‐mammals‐historical‐database.

[men70141-bib-0082] Notarbartolo‐di‐Sciara, G. , M. Zanardelli , M. Jahoda , S. Panigada , and S. Airoldi . 2003. “The Fin Whale Balaenoptera physalus (L. 1758) in the Mediterranean Sea.” Mammal Review 33, no. 2: 105–150.

[men70141-bib-0083] Ohland, D. P. , E. H. Harley , and P. B. Best . 1995. “Systematics of Cetaceans Using Restriction Site Mapping of Mitochondrial DNA.” Molecular Phylogenetics and Evolution 4, no. 1: 10–19. 10.1006/mpev.1995.1002. 7620632

[men70141-bib-0084] Othman, N. , H. Haris , Z. Fatin , et al. 2021. “A Review on Environmental DNA (eDNA) Metabarcoding Markers for Wildlife Monitoring Research. In IOP Conference Series.” Earth and Environmental Science 736, no. 1: 012054. 10.1088/1755-1315/736/1/012054.

[men70141-bib-0085] Panigada, V. , T. W. Bodey , A. Friedlaender , et al. 2024. “Targeting Fin Whale Conservation in the North‐Western Mediterranean Sea: Insights on Movements and Behaviour From Biologging and Habitat Modelling.” Royal Society Open Science 11, no. 3: 231783. 10.1098/rsos.231783. 38455994 PMC10915541

[men70141-bib-0086] Parent, G. J. , L. Montana , C. Bonnet , É. Parent , C. Sauvé , and A. P. St‐Pierre . 2025. “Genetic Monitoring Program for Beluga (Delphinapterus leucas) Harvested in the Nunavik and Nunavut (Belcher Islands) Regions.” Canadian Technical Report of Fisheries and Aquatic Sciences 3643: 34. 10.60825/erys-r351.

[men70141-bib-0087] Parra, G. J. 2023. “Australian Humpback Dolphin: Sousa sahulensis .” In Strahan's Mammals of Australia, edited by A. M. Baker and I. C. Gynther , 4th ed., 819–820. Bloomsbury.

[men70141-bib-0088] Parsons, K. M. , M. Everett , M. Dahlheim , and L. Park . 2018. “Water, Water Everywhere: Environmental DNA Can Unlock Population Structure in Elusive Marine Species.” Royal Society Open Science 5, no. 8: 180537. 10.1098/rsos.180537. 30225045 PMC6124077

[men70141-bib-0089] Parsons, K. M. , S. A. May , Z. Gold , et al. 2025. “Using eDNA to Supplement Population Genetic Analyses for Cryptic Marine Species: Identifying Population Boundaries for Alaska Harbour Porpoises.” Molecular Ecology 34, no. 5: e17563. 10.1111/mec.17563. 39450613 PMC11842950

[men70141-bib-0090] Peng, X. , Q. Li , Z. Cheng , and X. Huang . 2023. “The Geography of Genetic Data: Current Status and Future Perspectives.” Frontiers in Ecology and Evolution 11: 1112636. 10.3389/fevo.2023.1112636.

[men70141-bib-0139] Perrin, W. F. , E. D. Mitchell , J. G. Mead , et al. 1987. “Revision of the Spotted Dolphins, *Stenella* spp.” Marine Mammal Science 3, no. 2: 99–170.

[men70141-bib-0091] Piboon, P. , N. Kriengsakpichit , A. Poommouang , et al. 2022. “Relationship of Stranded Cetaceans in Thai Territorial Waters to Global Populations: Mitochondrial DNA Diversity of Cuvier's Beaked Whale, Indo Pacific Finless Porpoise, Pygmy Sperm Whale, and Dwarf Sperm Whale.” Science Progress 105, no. 2: 00368504221103776. 10.1177/00368504221103776. 35635263 PMC10450302

[men70141-bib-0092] Pierce, G. J. , M. A. Petitguyot , P. Gutierrez‐Muñoz , A. Fariñas‐Bermejo , D. Fernández‐Fernández , and S. Dolman . 2024. “Chapter an Endangered Population of Harbour Porpoise Phocoena phocoena Hidden in Plain Sight: Biology, Ecology and Conservation of the Iberian Porpoise.” In Oceanography and Marine Biology. Taylor & Francis. 10.1201/9781003477518-1.

[men70141-bib-0093] Pitman, R. L. , and P. Ensor . 2003. “Three Forms of Killer Whales (Orcinus orca) in Antarctic Waters.” Journal of Cetacean Research and Management 5, no. 2: 131–139. 10.47536/jcrm.v5i2.813.

[men70141-bib-0094] Pitogo, K. M. E. 2025. “Gaps and Biases in Vertebrate Wildlife Genetics From a Global Biodiversity Hotspot.” Environmental Conservation 52, no. 3: 127–138. 10.1017/S0376892925000141.

[men70141-bib-0095] Pompa, S. 2011. “Global Distribution and Conservation of Marine Mammals.” Proceedings of the National Academy of Sciences 108, no. 33: 13491–13496. 10.1073/pnas.1101525108. PMC3158205 21808012

[men70141-bib-0096] Ratnasingham, S. , and P. D. Hebert . 2007. “BOLD: The Barcode of Life Data System (Http://Www. barcodingl*ife. Org*).” Molecular Ecology Notes 7, no. 3: 355–364. 10.1111/j.1471-8286.2007.01678.x. 18784790 PMC1890991

[men70141-bib-0097] Reeves, R. , and G. Notarbartolo di Sciara . 2006. The Status and Distribution of Cetaceans in the Black Sea and Mediterranean Sea, 137. IUCN Centre for Mediterranean Cooperation.

[men70141-bib-0141] Robinson, C. V. , K. Dracott , R. D. Glover , A. Warner , and A. Migneault . 2024. “DNA from Dives: Species detection of humpback whales (*Megaptera novaeangliae*) from flukeprint Edna.” Environmental DNA 6, no. 2: e524. 10.1002/edn3.524.

[men70141-bib-0098] Roman, J. , and J. A. Estes . 2018. “Ecology.” In Encyclopedia of Marine Mammals, edited by B. Würsig , J. G. M. Thewissen , and K. Kovacs , 299–303. Elsevier. 10.1016/B978-0-12-804327-1.00114-X.

[men70141-bib-0099] Rosel, P. E. , B. L. Taylor , B. L. Hancock‐Hanser , et al. 2017. “A Review of Molecular Genetic Markers and Analytical Approaches That Have Been Used for Delimiting Marine Mammal Subspecies and Species.” Marine Mammal Science 33, no. S1: 56–75. 10.1111/mms.12412.

[men70141-bib-0100] Rosel, P. E. , L. A. Wilcox , T. K. Yamada , and K. D. Mullin . 2021. “A New Species of Baleen Whale (Balaenoptera) From the Gulf of Mexico, With a Review of Its Geographic Distribution.” Marine Mammal Science 37, no. 2: 577–610. 10.1111/mms.12776.

[men70141-bib-0101] Sard, N. M. , S. J. Herbst , L. Nathan , et al. 2019. “Comparison of Fish Detections, Community Diversity, and Relative Abundance Using Environmental DNA Metabarcoding and Traditional Gears.” Environmental DNA 1, no. 4: 368–384. 10.1002/edn3.38.

[men70141-bib-0102] Sasaki, T. , M. Nikaido , H. Hamilton , et al. 2005. “Mitochondrial Phylogenetics and Evolution of Mysticete Whales.” Systematic Biology 54, no. 1: 77–90. 10.1080/10635150590905939. 15805012

[men70141-bib-0103] Sayers, E. W. , J. Beck , E. E. Bolton , D. Bourexis , J. R. Brister , and K. Canese . 2021. “Database Resources of the National Center for Biotechnology Information.” Nucleic Acids Research 49, no. D1: D10–D17. 10.1093/nar/gkx1095. 33095870 PMC7778943

[men70141-bib-0104] Schoch, C. L. , S. Ciufo , M. Domrachev , et al. 2020. “NCBI Taxonomy: A Comprehensive Update on Curation, Resources and Tools.” Database 2020: baaa062. 10.1093/database/baaa062. 32761142 PMC7408187

[men70141-bib-0105] Sigsgaard, E. E. , M. R. Jensen , I. E. Winkelmann , P. R. Møller , M. M. Hansen , and P. F. Thomsen . 2020. “Population‐Level Inferences From Environmental DNA—Current Status and Future Perspectives.” Evolutionary Applications 13, no. 2: 245–262. 10.1111/eva.12882. 31993074 PMC6976968

[men70141-bib-0106] Sigsgaard, E. E. , I. B. Nielsen , S. S. Bach , E. D. Lorenzen , D. P. Robinson , and S. W. Knudsen . 2016. “Population Characteristics of a Large Whale Shark Aggregation Inferred From Seawater Environmental DNA.” Nature Ecology & Evolution 1, no. 1: 0004. 10.1038/s41559-016-0004. 28812572

[men70141-bib-0107] Skovrind, M. , J. A. S. Castruita , J. Haile , et al. 2019. “Hybridization Between Two High Arctic Cetaceans Confirmed by Genomic Analysis.” Scientific Reports 9, no. 1: 7729. 10.1038/s41598-019-44038-0. 31221994 PMC6586676

[men70141-bib-0108] Šmíd, J. 2022. “Geographic and Taxonomic Biases in the Vertebrate Tree of Life.” Journal of Biogeography 49, no. 12: 2120–2129. 10.1111/jbi.14491.

[men70141-bib-0109] Smith, B. D. , D. Wang , G. T. Braulik , R. Reeves , K. Zhou , and J. Barlow . 2017. “ Lipotes vexillifer .” IUCN Red List of Threatened Species 2017: e.T12119A50362206. 10.2305/IUCN.UK.2017-3.RLTS.T12119A50362206.en.

[men70141-bib-0110] Smith, D. R. 2016. “The Past, Present and Future of Mitochondrial Genomics: Have We Sequenced Enough mtDNAs?” Briefings in Functional Genomics 15, no. 1: 47–54. 10.1093/bfgp/elv027. 26117139 PMC4812591

[men70141-bib-0111] Society for Marine Mammalogy . 2025. “List of marine mammal species and subspecies.” https://marinemammalscience.org/science‐and‐publications/list‐marine‐mammal‐species‐subspecies/.

[men70141-bib-0112] Suarez‐Bregua, P. , M. Álvarez‐González , K. M. Parsons , J. Rotllant , G. J. Pierce , and C. Saavedra . 2022. “Environmental DNA (eDNA) for Monitoring Marine Mammals: Challenges and Opportunities.” Frontiers in Marine Science 9: 987774. 10.3389/fmars.2022.987774.

[men70141-bib-0113] Swartz, S. L. 2018. “Gray whale: Eschrichtius robustus .” In Encyclopedia of Marine Mammals, 422–428. Academic Press. 10.1016/B978-0-12-804327-1.00140-0.

[men70141-bib-0114] Takahashi, M. , T. G. Frøslev , J. Paupério , B. Thalinger , K. Klymus , and C. C. Helbing . 2025. “A Metadata Checklist and Data Formatting Guidelines to Make eDNA FAIR (Findable, Accessible, Interoperable, and Reusable).” Environmental DNA 7, no. 3: e70100. 10.1002/edn3.70100.

[men70141-bib-0115] Teixeira, M. A. , E. Aylagas , J. K. Pearman , and S. Carvalho . 2025. “Gaps and Data Ambiguities in DNA Reference Libraries: A Limiting Factor for Molecular‐Based Biodiversity Assessments Using Annelids as a Case Study.” Ecology and Evolution 15, no. 6: e71544. 10.1002/ece3.71544. 40546907 PMC12178944

[men70141-bib-0116] Thiele, D. , E. T. Chester , and P. C. Gill . 2000. “Cetacean Distribution Off Eastern Antarctica (80–150 E) During the Austral Summer of 1995/1996.” Deep Sea Research Part II: Topical Studies in Oceanography 47, no. 12–13: 2543–2572. 10.1016/S0967-0645(00)00035-7.

[men70141-bib-0117] Tyack, P. L. , M. Johnson , N. A. Soto , A. Sturlese , and P. T. Madsen . 2006. “Extreme Diving of Beaked Whales.” Journal of Experimental Biology 209, no. 21: 4238–4253. 10.1242/jeb.02505. 17050839

[men70141-bib-0118] Tydecks, L. , J. M. Jeschke , M. Wolf , G. Singer , and K. Tockner . 2018. “Spatial and Topical Imbalances in Biodiversity Research.” PLoS One 13, no. 7: e0199327. 10.1371/journal.pone.0199327. 29975719 PMC6033392

[men70141-bib-0119] Ushio, M. , S. Ozawa , S. I. Oka , T. Sado , R. O. Kisero , and L. Porter . 2025. “μCeta: A Set of Cetacean‐Specific Primers for Environmental DNA Metabarcoding With Minimal Amplification of Non‐Target Vertebrates.” Environmental DNA 7, no. 5: e70193. 10.1002/edn3.70193.

[men70141-bib-0120] Valsecchi, E. , A. Arcangeli , R. Lombardi , et al. 2021. “Ferries and Environmental DNA: Underway Sampling From Commercial Vessels Provides New Opportunities for Systematic Genetic Surveys of Marine Biodiversity.” Frontiers in Marine Science 8: 704786. 10.1101/2021.02.13.431078.

[men70141-bib-0121] Valsecchi, E. , J. Bylemans , S. J. Goodman , et al. 2020. “Novel Universal Primers for Metabarcoding eDNA Surveys of Marine Mammals and Other Marine Vertebrates.” Environmental DNA 2, no. 4: 460–476. 10.1002/edn3.72.

[men70141-bib-0122] van den Burg, M. P. , and D. R. Vieites . 2023. “Bird Genetic Databases Need Improved Curation and Error Reporting to NCBI.” Ibis 165, no. 2: 472–481. 10.1111/ibi.13143.

[men70141-bib-0123] Vieira, P. E. , A. S. Lavrador , M. I. Parente , et al. 2021. “Gaps in DNA Sequence Libraries for Macaronesian Marine Macroinvertebrates Imply Decades Till Completion and Robust Monitoring.” Diversity and Distributions 27, no. 10: 2003–2015. 10.1111/ddi.13305.

[men70141-bib-0124] Wang, Z. , X. Liu , D. Liang , Q. Wang , L. Zhang , and P. Zhang . 2023. “VertU: Universal Multilocus Primer Sets for eDNA Metabarcoding of Vertebrate Diversity, Evaluated by Both Artificial and Natural Cases.” Frontiers in Ecology and Evolution 11: 1164206. 10.3389/fevo.2023.1164206.

[men70141-bib-0125] Weir, C. R. , and T. Collins . 2015. “A Review of the Geographical Distribution and Habitat of the Atlantic Humpback Dolphin (Sousa teuszii).” In Advances in Marine Biology, edited by T. A. Jefferson and B. C. Curry , vol. 72, 79–117. Academic Press. 10.1016/bs.amb.2015.08.001 26555623

[men70141-bib-0126] Weir, C. R. , K. Van Waerebeek , T. A. Jefferson , and T. Collins . 2011. “West Africa's Atlantic Humpback Dolphin (Sousa teuszii): Endemic, Enigmatic and Soon Endangered?” African Zoology 46, no. 1: 1–17. 10.1080/15627020.2011.11407473.

[men70141-bib-0127] Weitemier, K. , B. E. Penaluna , L. L. Hauck , L. J. Longway , T. Garcia , and R. Cronn . 2021. “Estimating the Genetic Diversity of Pacific Salmon and Trout Using Multigene eDNA Metabarcoding.” Molecular Ecology 30, no. 20: 4970–4990. 10.1111/mec.15811. 33594756 PMC8597136

[men70141-bib-0128] Wickham, H. , W. Chang , and M. H. Wickham . 2016. “Package ‘ggplot2’: Create elegant data visualisations using the grammar of graphics (Version 2, pp. 1–189).”

[men70141-bib-0129] Wickham, H. , and M. H. Wickham . 2021. “Package ‘forcats’.”

[men70141-bib-0130] Wickham, H. , M. H. Wickham , and RColorBrewer, I . 2016. “Package ‘scales’ (R package version 1.0).”

[men70141-bib-0131] Wilcox, T. M. , K. S. McKelvey , M. K. Young , et al. 2013. “Robust Detection of Rare Species Using Environmental DNA: The Importance of Primer Specificity.” PLoS One 8, no. 3: e59520. 10.1371/journal.pone.0059520. 23555689 PMC3608683

[men70141-bib-0132] Wilkinson, M. D. , M. Dumontier , I. J. Aalbersberg , G. Appleton , M. Axton , and A. Baak . 2016. “The FAIR Guiding Principles for Scientific Data Management and Stewardship.” Scientific Data 3, no. 1: 1–9. 10.1038/sdata.2016.18. PMC4792175 26978244

[men70141-bib-0133] Winter, D. J. 2017. “Rentrez: An R Package for the NCBI eUtils API.” PeerJ Preprints: e3179v2. 10.7287/peerj.preprints.3179v2.

[men70141-bib-0134] Wiszniewski, J. , L. B. Beheregaray , S. J. Allen , and L. M. Möller . 2010. “Environmental and Social Influences on the Genetic Structure of Bottlenose Dolphins (Tursiops aduncus) in Southeastern Australia.” Conservation Genetics 11: 1405–1419. 10.1007/s10592-009-9968-z.

[men70141-bib-0135] Wolf, M. , K. Zapf , D. K. Gupta , M. Hiller , Ú. Árnason , and A. Janke . 2023. “The Genome of the Pygmy Right Whale Illuminates the Evolution of Rorquals.” BMC Biology 21, no. 1: 79. 10.1186/s12915-023-01579-1. 37041515 PMC10091562

[men70141-bib-0136] WoRMS (World Register of Marine Species) . 2025. “World Cetacea Database.” https://www.marinespecies.org/cetacea/aphia.php?id=2688.

[men70141-bib-0137] Yang, L. , Z. Tan , D. Wang , et al. 2014. “Species Identification Through Mitochondrial rRNA Genetic Analysis.” Scientific Reports 4, no. 1: 4089. 10.1038/srep04089. 24522485 PMC5379257

[men70141-bib-0138] Yoshitake, K. , K. Yanagisawa , Y. Sugimoto , et al. 2023. “Pilot Study of a Comprehensive Resource Estimation Method From Environmental DNA Using Universal D‐Loop Amplification Primers.” Functional & Integrative Genomics 23, no. 2: 96. 10.1007/s10142-023-01013-3. 36947319 PMC10033627

