# Supplementary material for: Navigating Cetacean Mitochondrial Genome Data: Identifying Coverage and Deficiencies in Public Repositories
Source: Mol Ecol Resour. 2026 Apr 16;26(3):e70141. doi: 10.1111/1755-0998.70141 (PMC13087493; doi:10.1111/1755-0998.70141)

Navigating Cetacean Mitochondrial Genome Data: Identifying Coverage and Deficiencies in Public Repositories

Luís Afonso^1,2,3,*^, Alessandro Lagrotteria^1,4,5^, Ana Sofia Lavrador^1^, Paula Suarez-Bregua^6^, Miguel Álvarez-González^6^, Raul Valente^1,7^, Mads Reinholdt Jensen^8^, Camilo Saavedra^6^, Daniel Kumazawa Morais^8^, Graham J. Pierce^3^, Catarina Magalhães^1,7^, Ana Mafalda Correia^1^

^1^CIIMAR/CIIMAR-LA - Interdisciplinary Centre of Marine and Environmental Research, University of Porto 4450-208, Matosinhos, Portugal.

^2^ICBAS-UP - School of Medicine and Biomedical Sciences, University of Porto 4050-313, Porto, Portugal.

^3^IIM-CSIC - Institute of Marine Research of the Spanish National Research Council, 36208, Vigo, Pontevedra, Spain

^4^CNR-IRET - Research Institute on Terrestrial Ecosystems of the Italian National Research Council, 50019 Florence, Italy.

^5^DBIOS - Department of Life Sciences and Systems Biology, University of Turin, 10123, Turin, Italy

^6^IEO-CSIC - Spanish Institute of Oceanography of the Spanish National Research Council, 36390, Vigo, Pontevedra, Spain.

^7^FCUP - Faculty of Sciences of the University of Porto, 4169-007, Porto, Portugal.

^8^Norwegian College of Fishery Science, UiT—The Arctic University of Norway, 9019, Tromsø, Norway

*Corresponding author: [lafonso@ciimar.up.pt](mailto:lafonso@ciimar.up.pt)

**Figure S1 (A, B, C, D) - Marker abundance across cetacean species.**
Proportional distribution of unique accessions across five mitochondrial markers (D-loop, COI, Cytb, 16S rRNA, and 12S rRNA) for each cetacean taxon included in the dataset. Marker composition is shown as stacked proportions, representing the relative contribution of each marker within taxa and allowing comparison independent of total sequence abundance. These species-level profiles expand upon the family-level overview presented in Figure 1 of the main text and provide finer resolution of marker representation within individual taxa. Panels are organized by cetacean family and grouped by suborder (Odontoceti and Mysticeti) to maintain taxonomic structure and improve visualization across the full dataset.

**A)
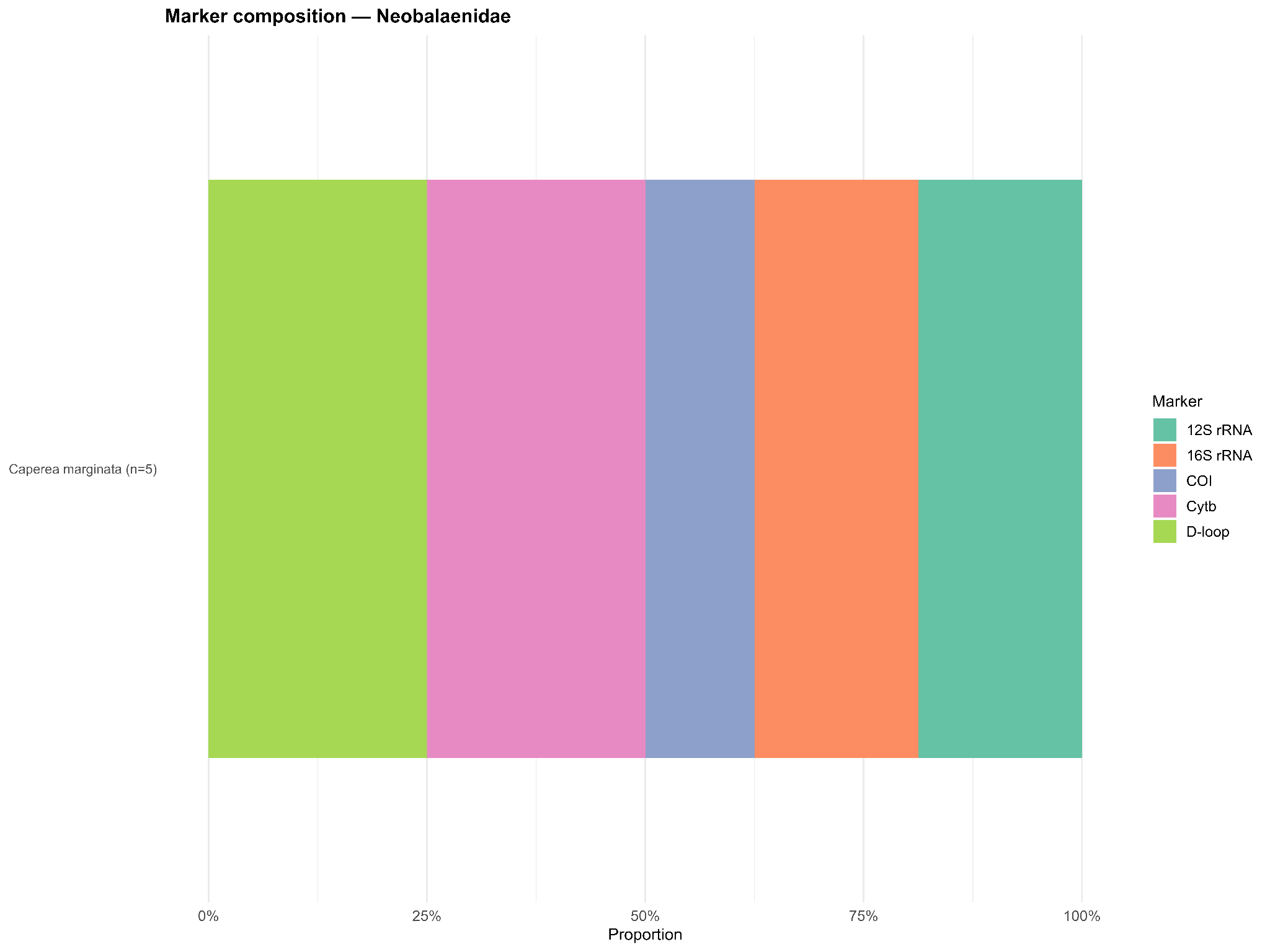
**

**B)
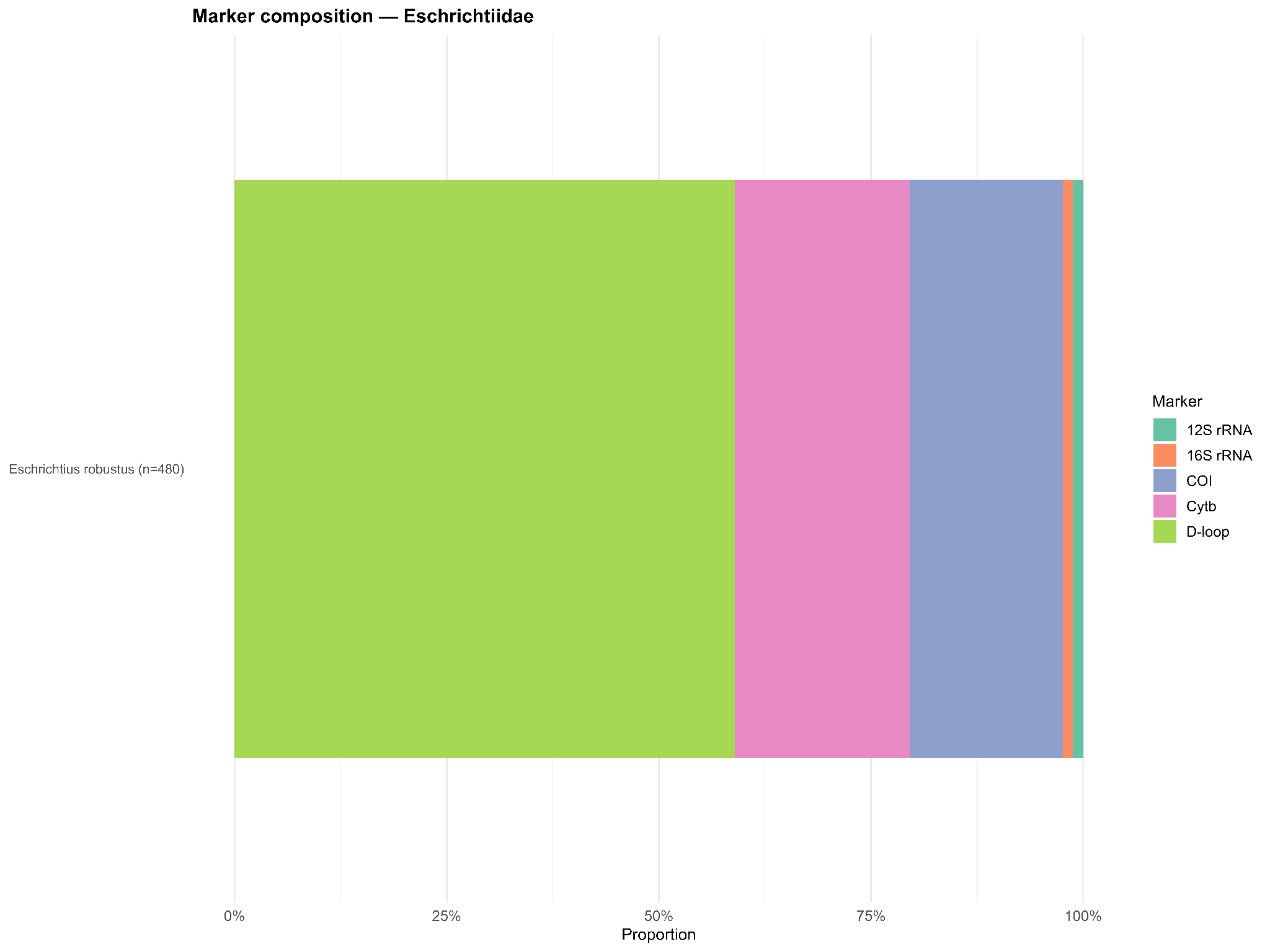
**

**C)
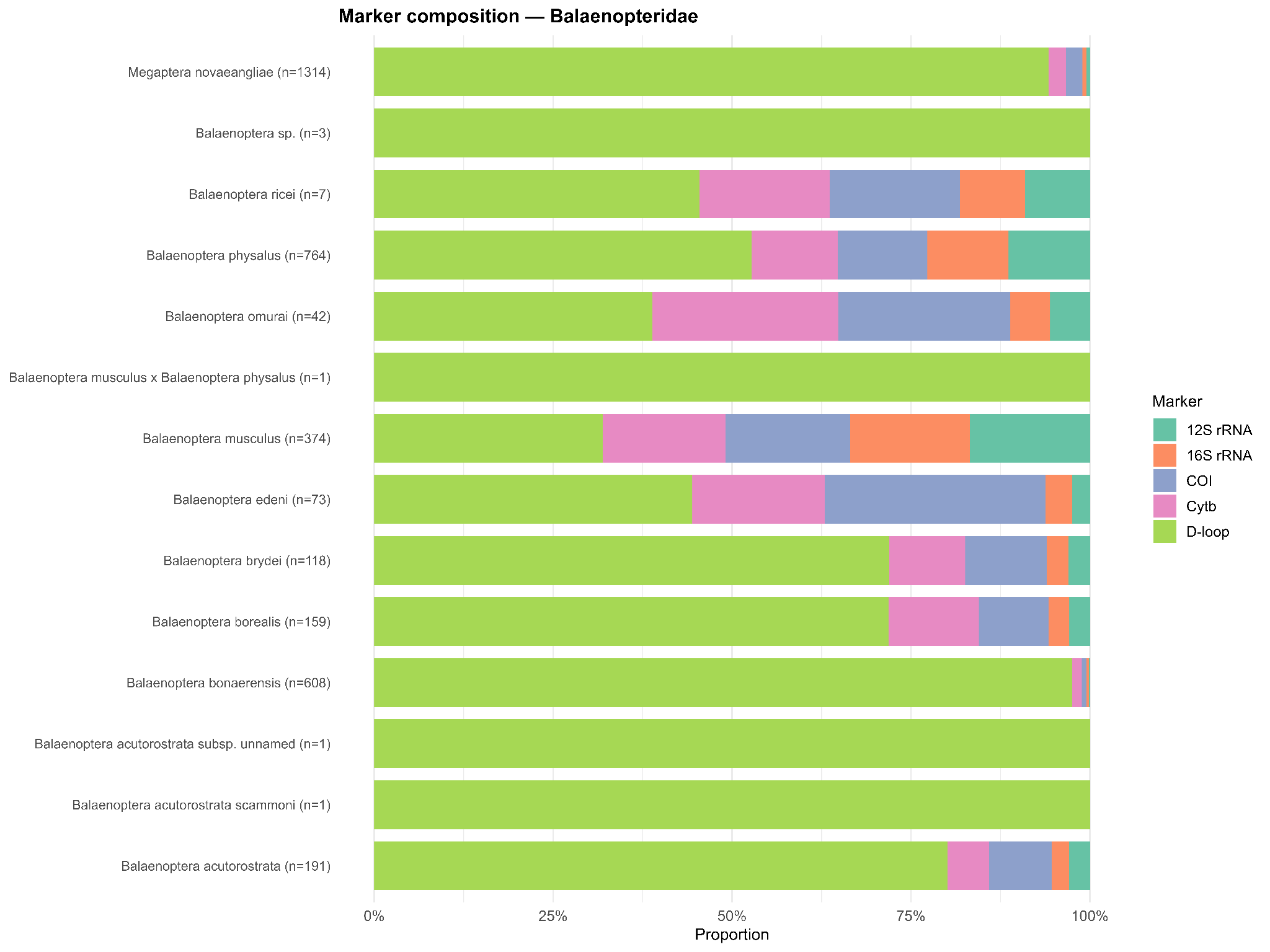
**

**D)
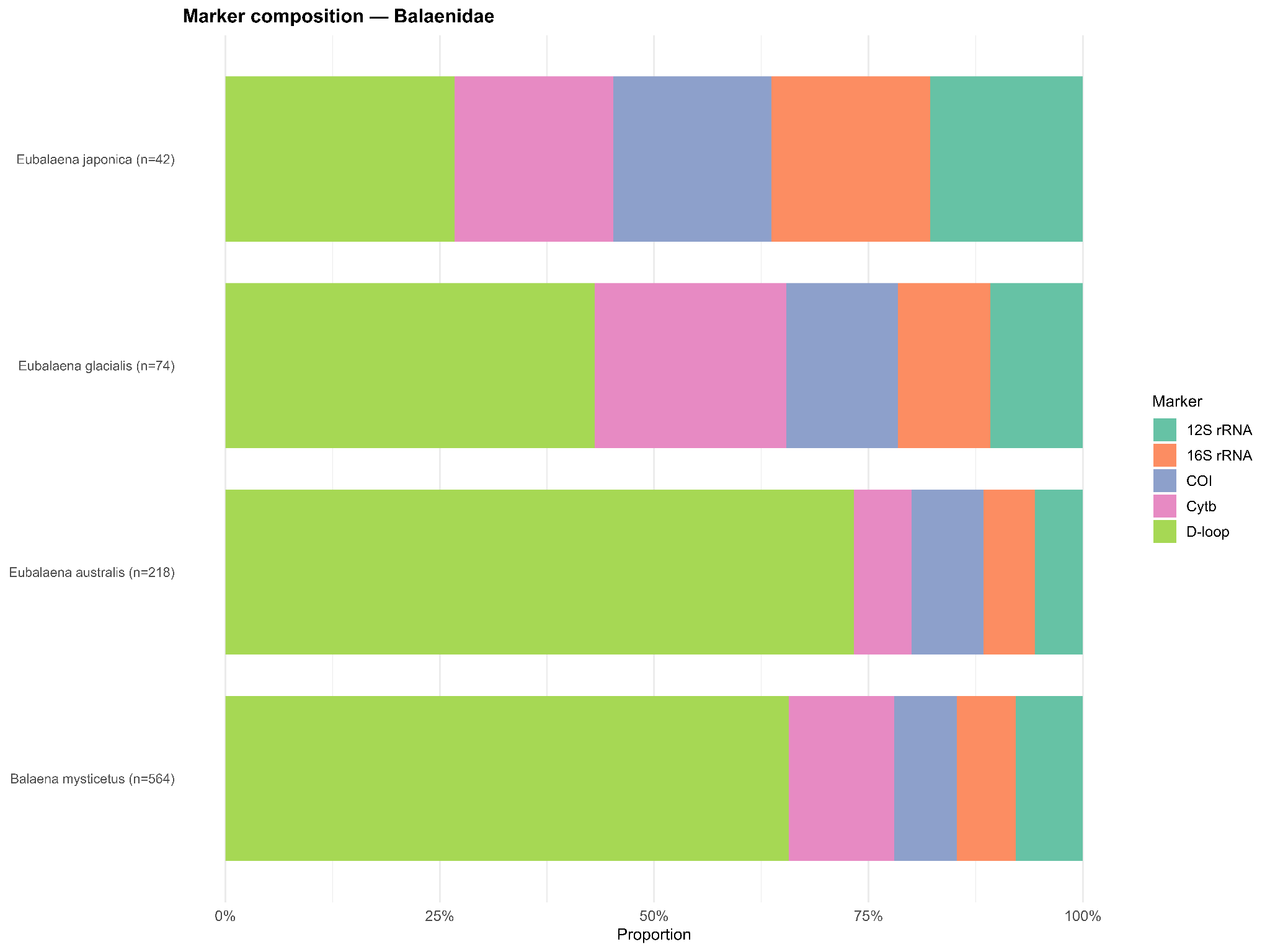
**

**E)
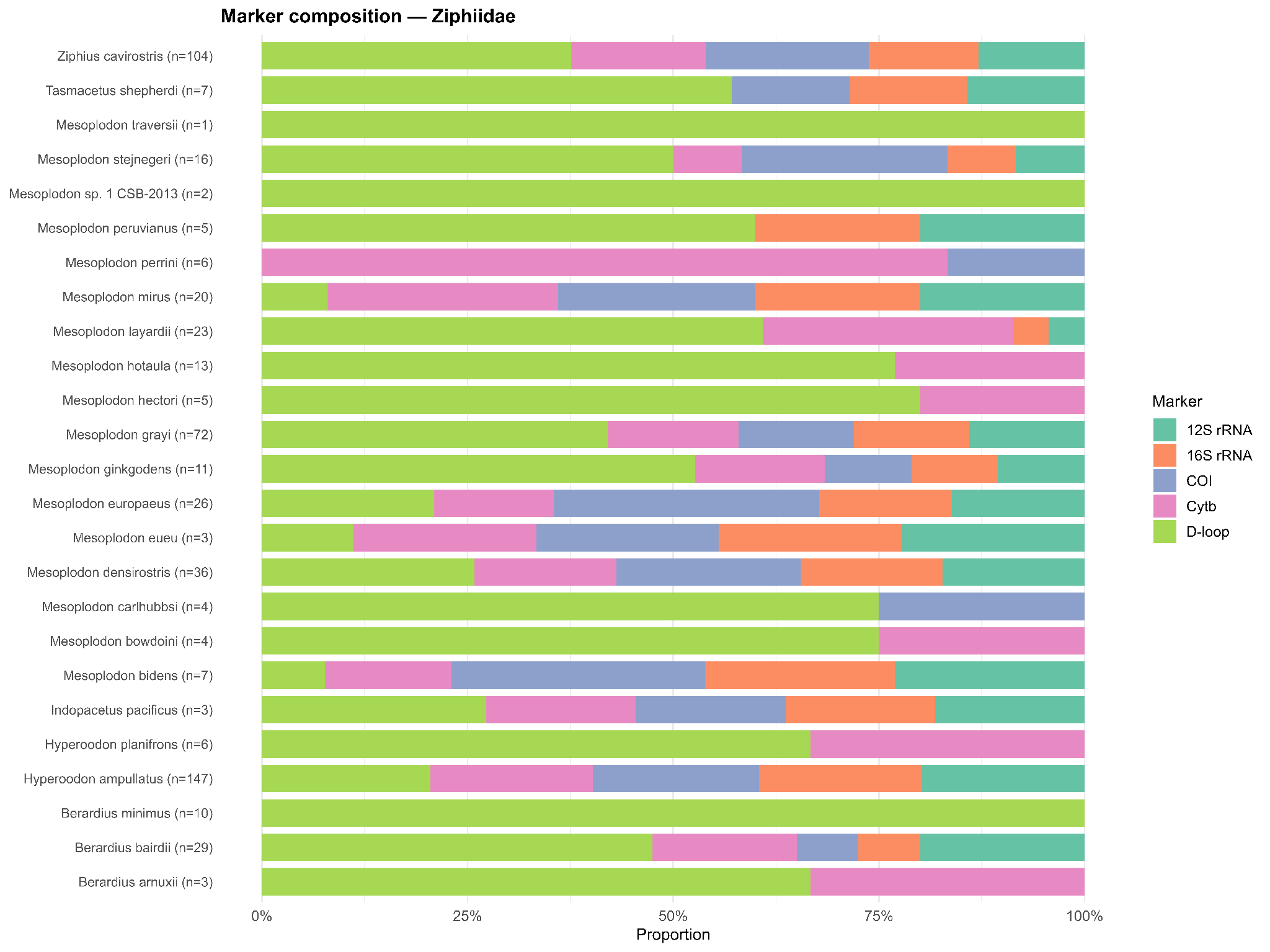
**

**F)
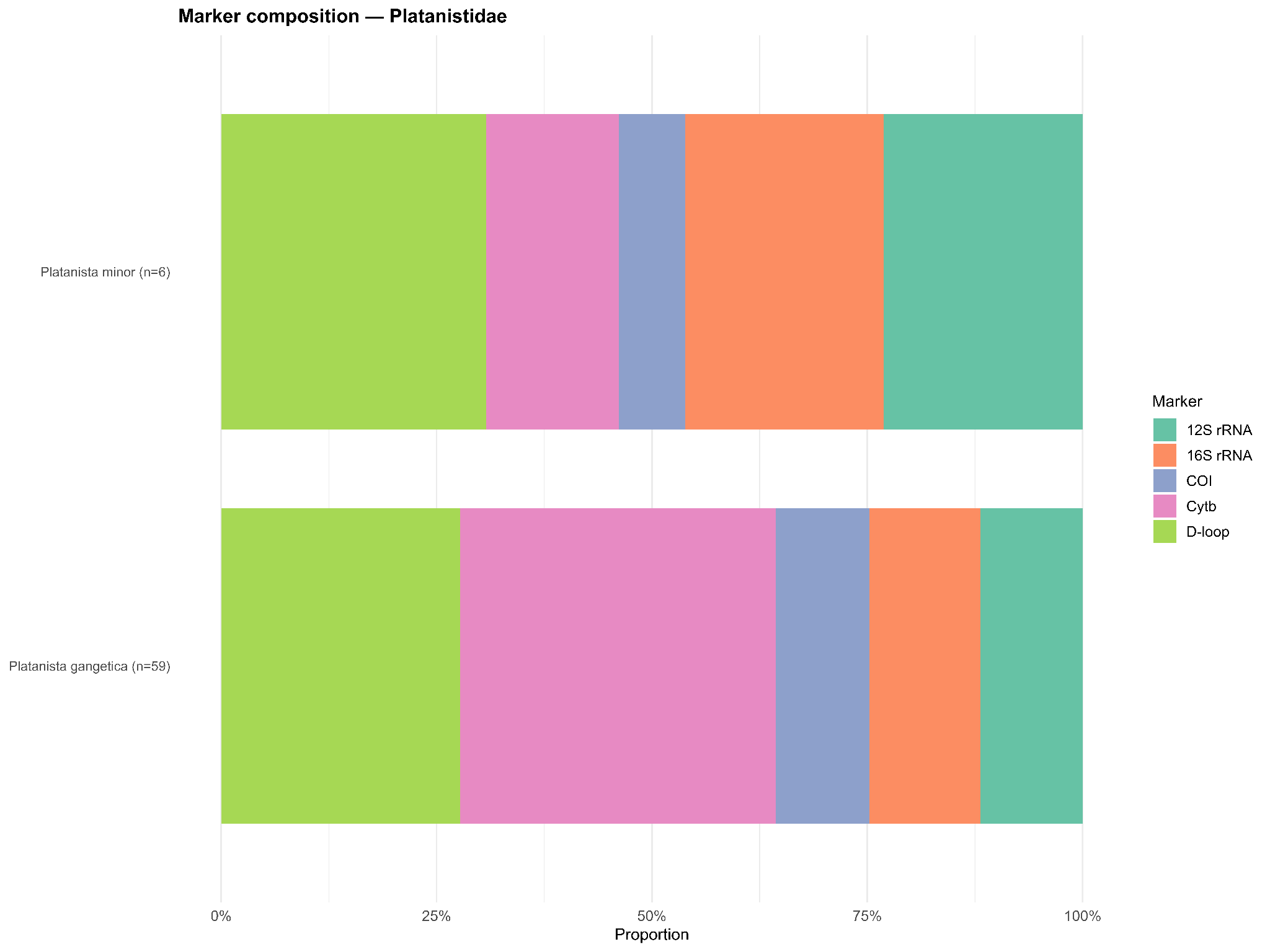
**

**G)
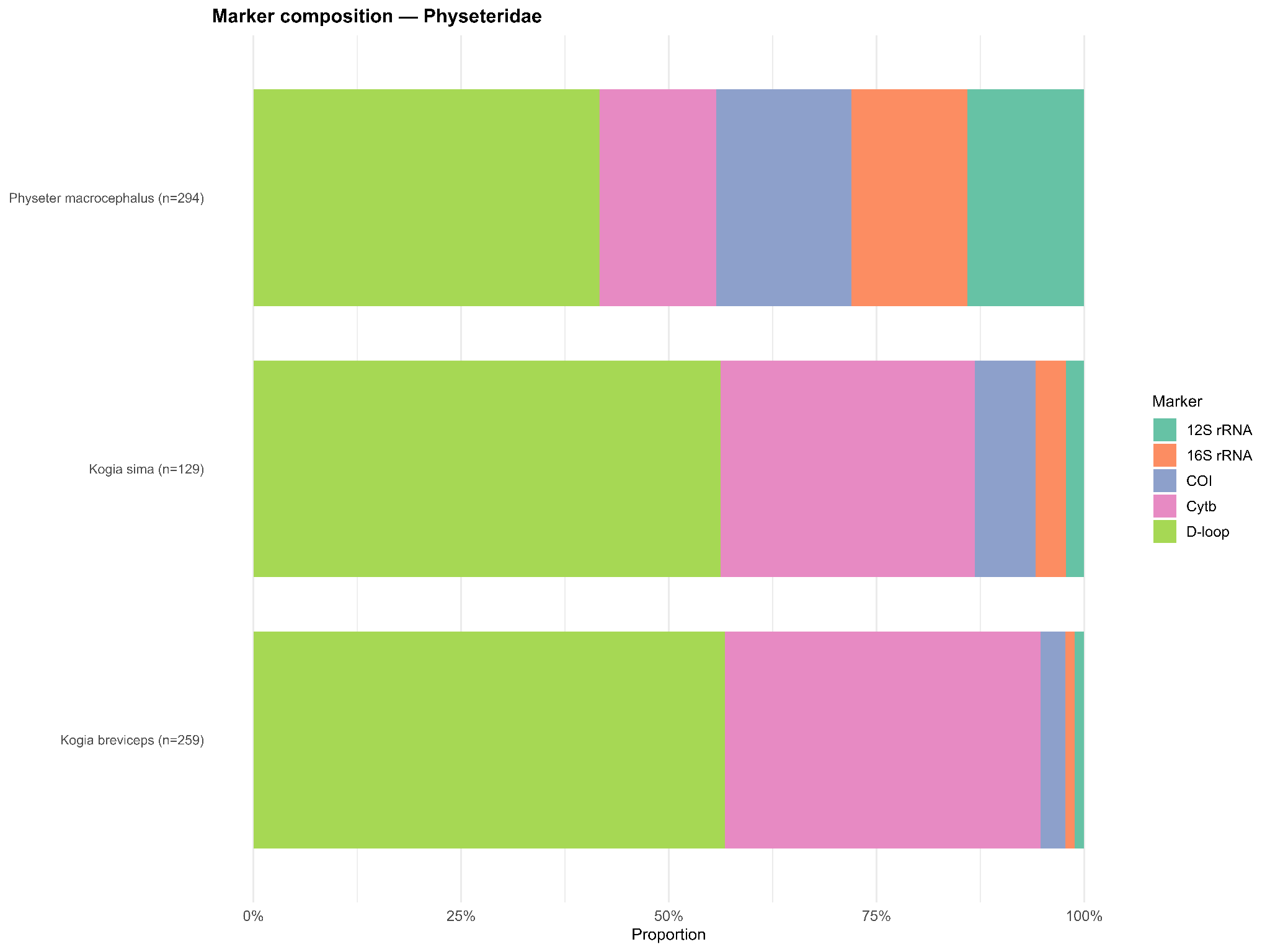
**

**H)
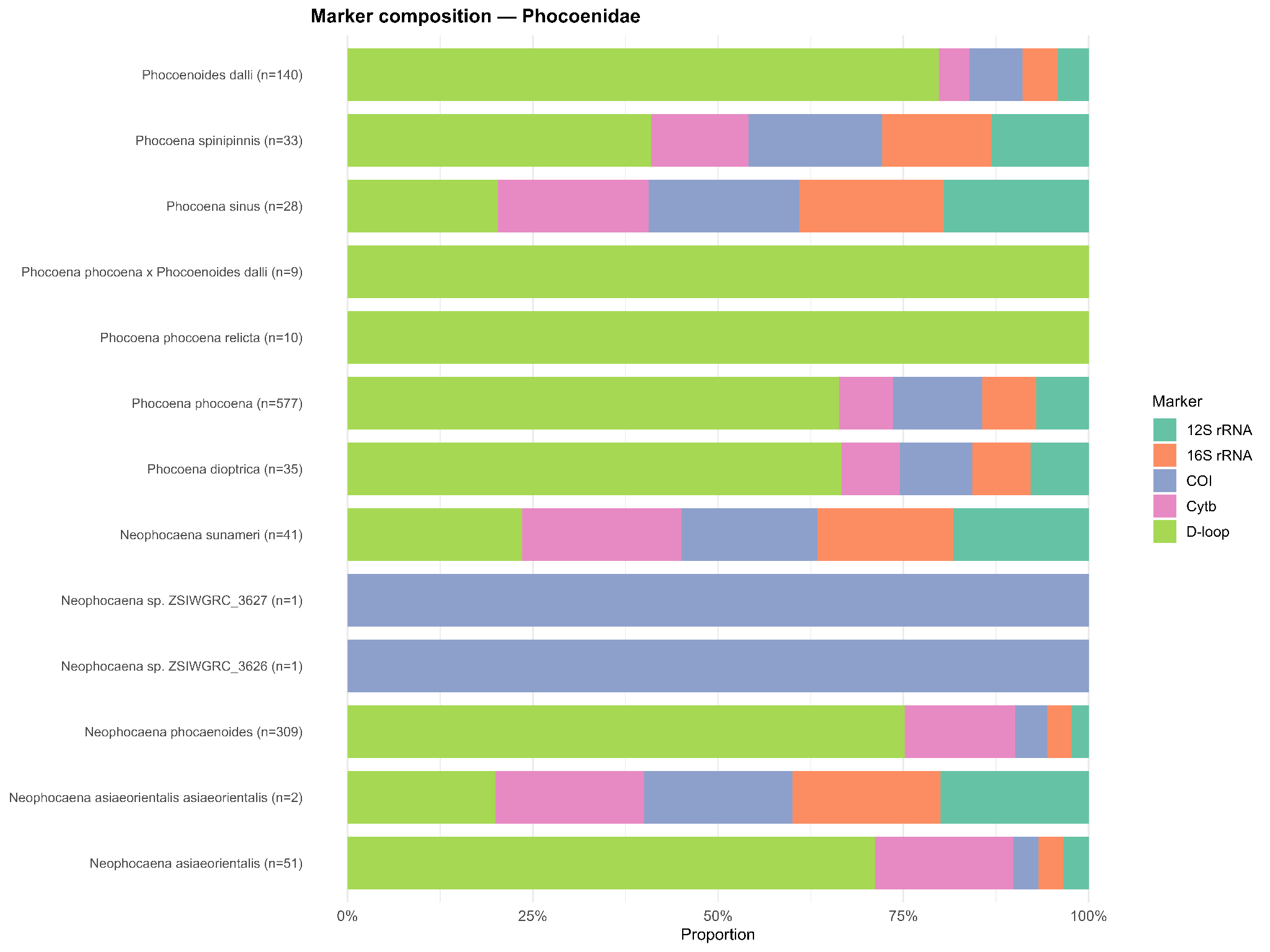
**

**I)
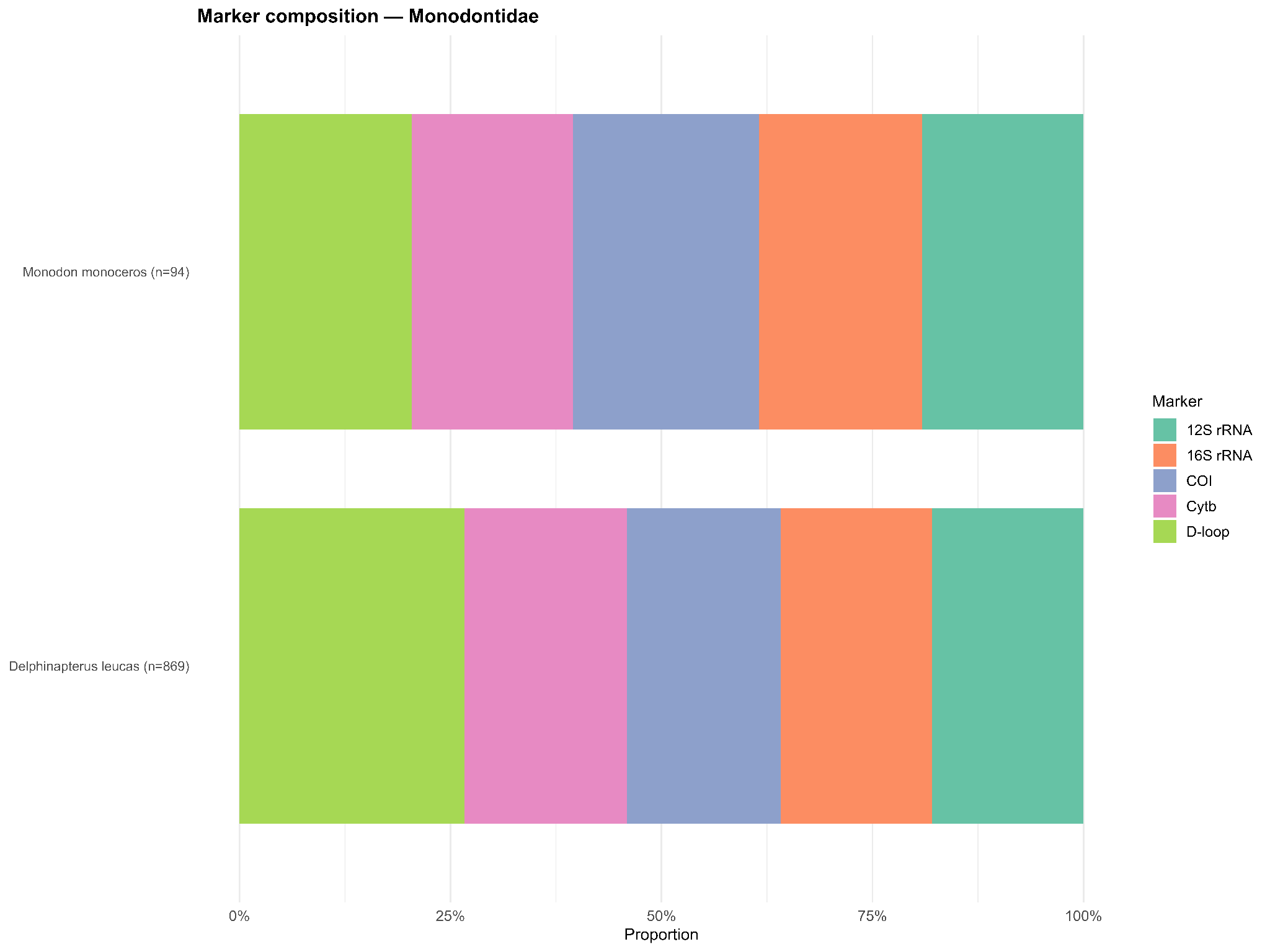
**

**J)
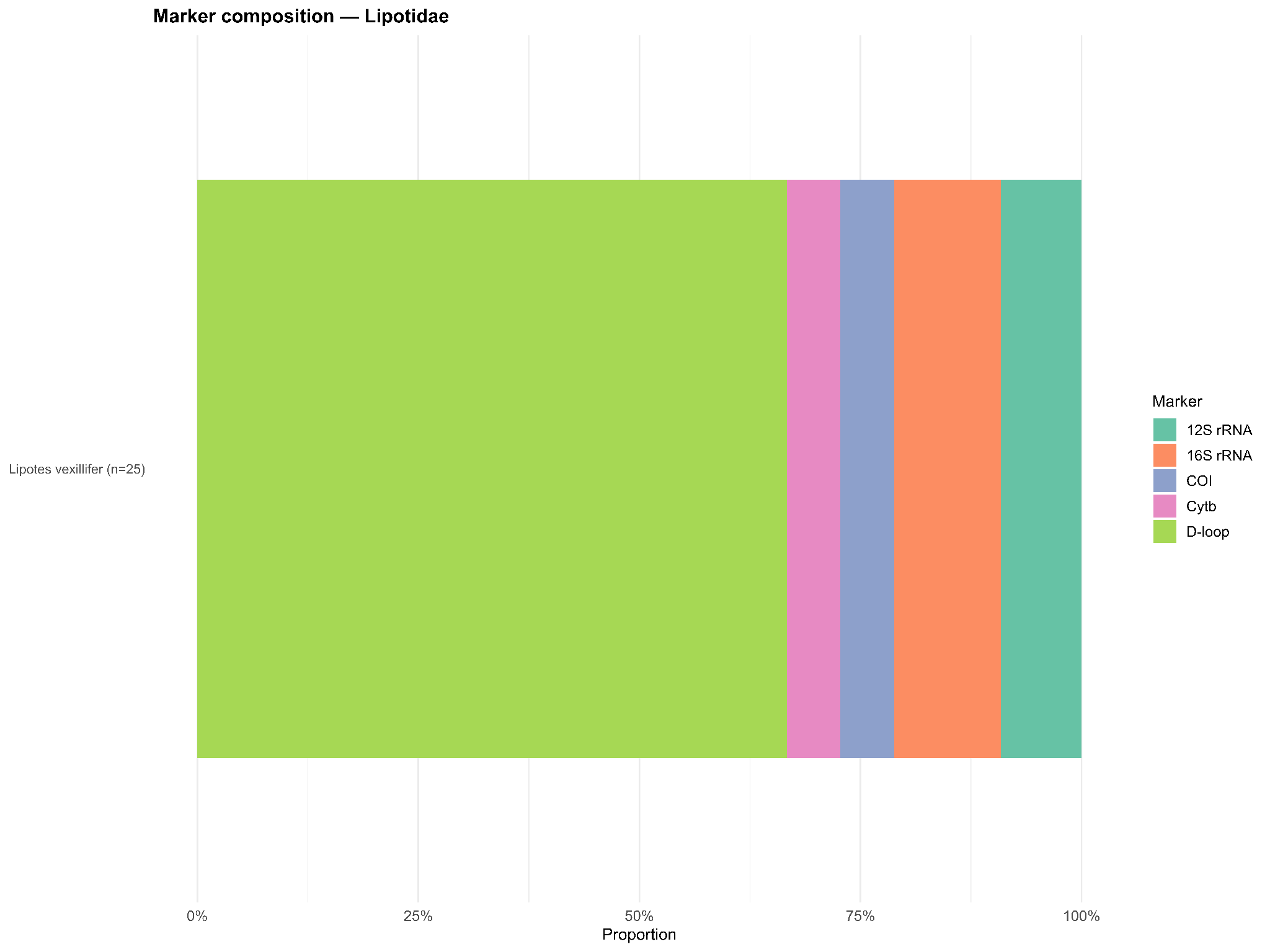
**

**K)
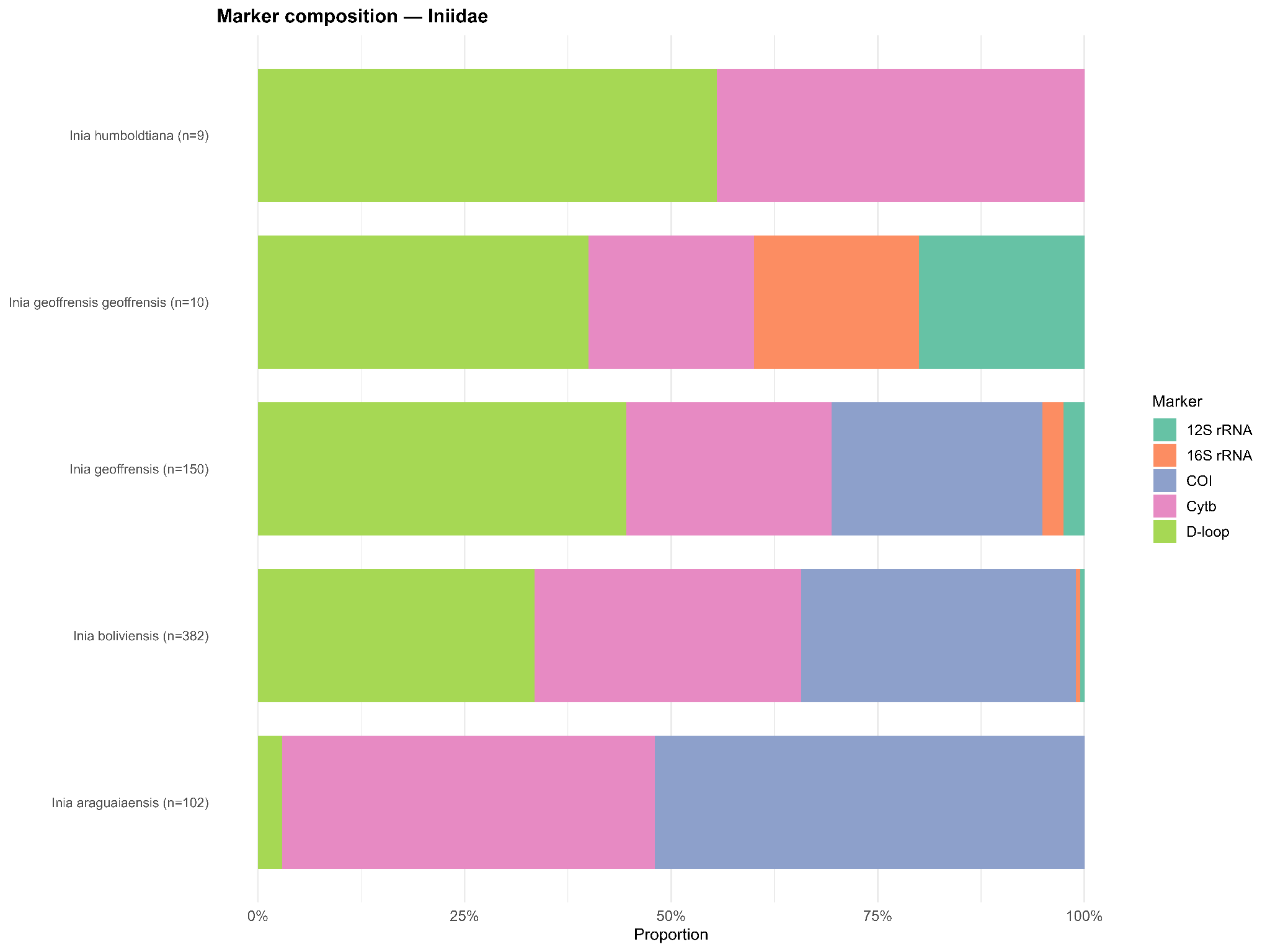
**

**L)
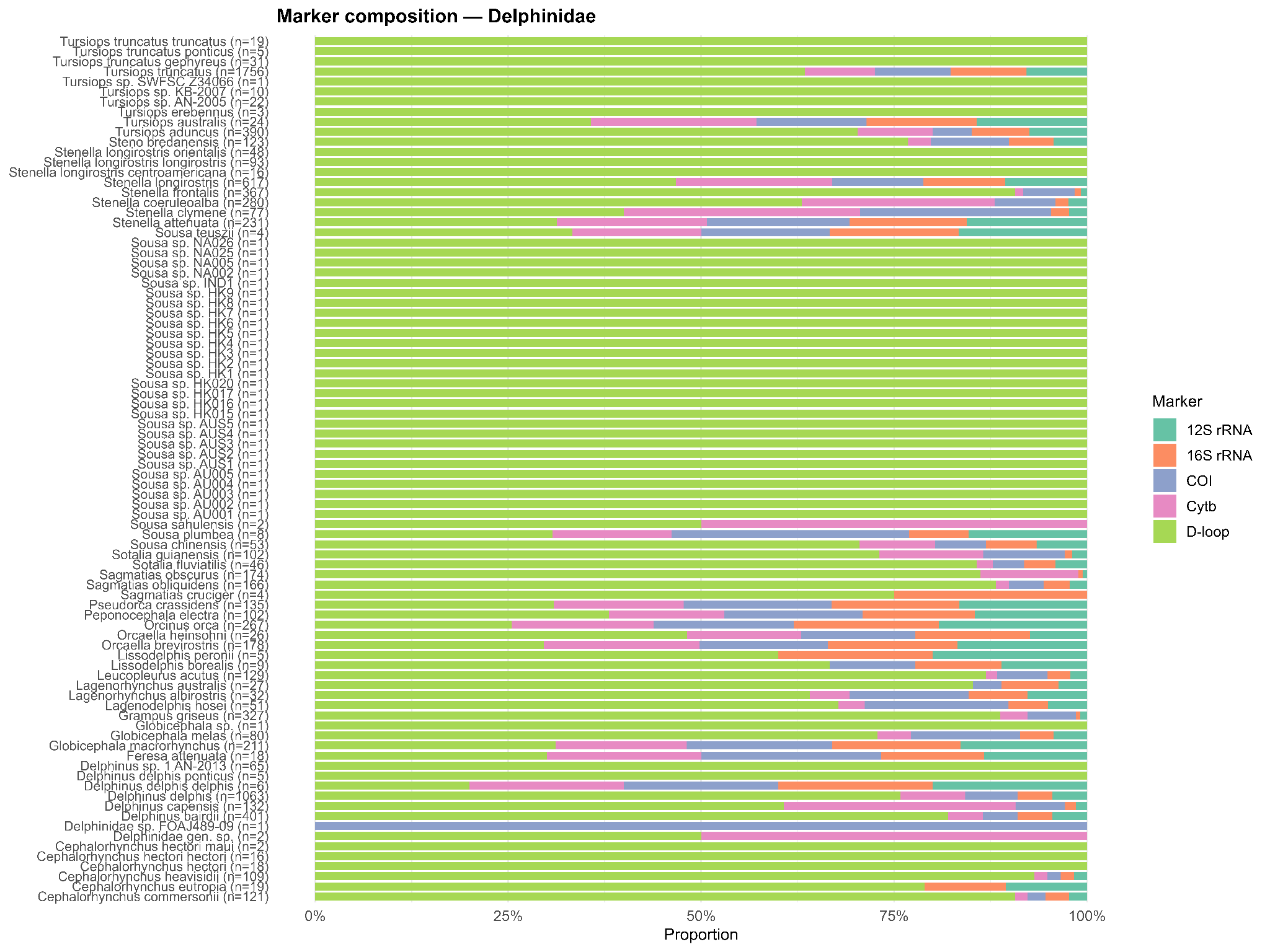
**

**Figure S2 - Geographic distribution of cumulative sequences across cetacean families.**
Maps show the distribution of records across 40 geographic clusters (see Methods). Individualized family-level patterns complement the global overview presented in Figure 4B of the main text.

**A) Balaenidae

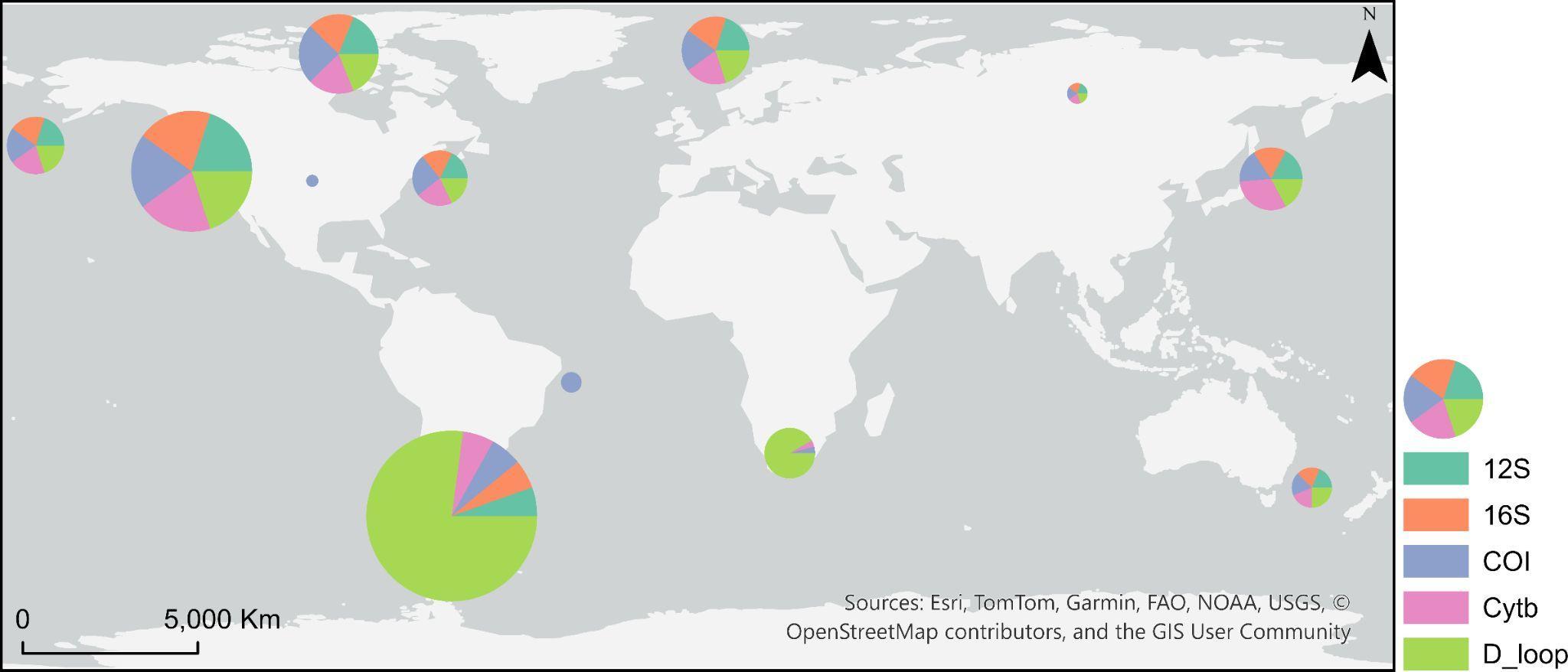
**

**B) Balaenopteridae

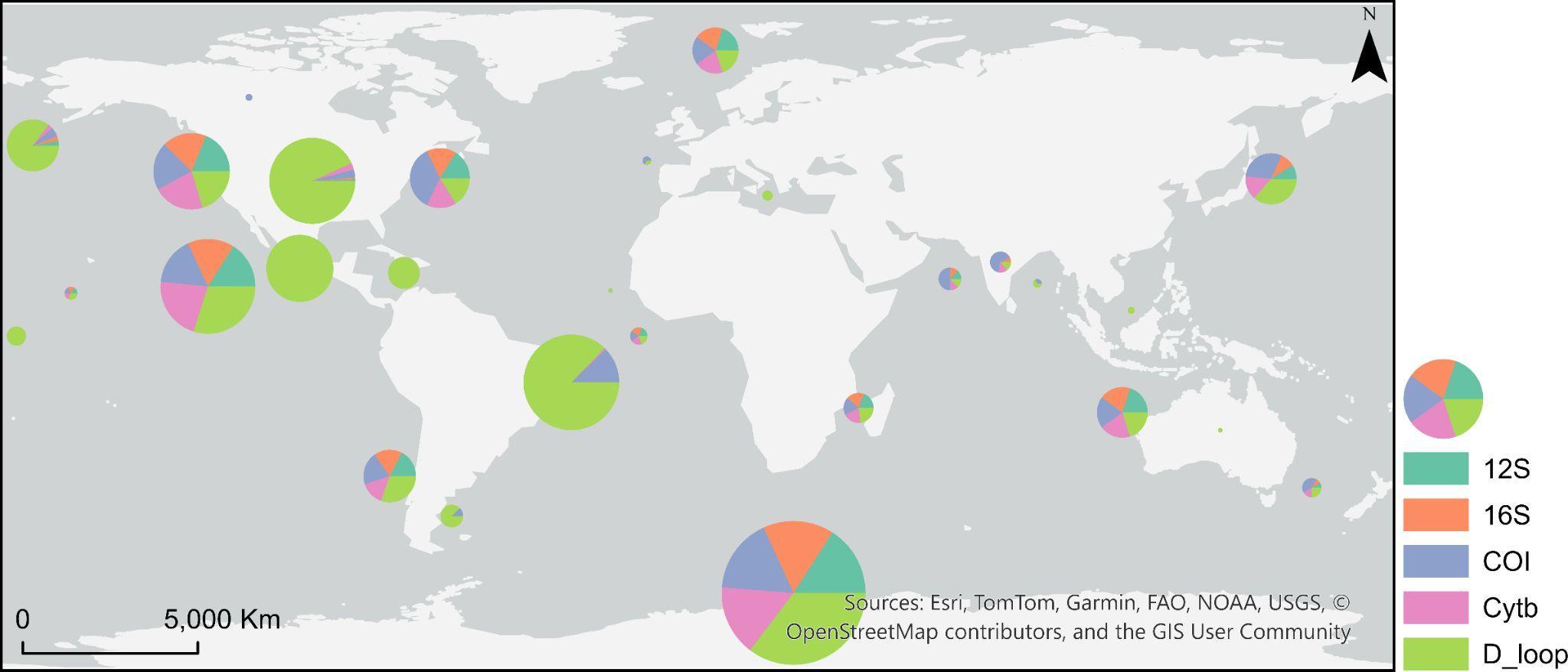
**

**C) Delphinidae

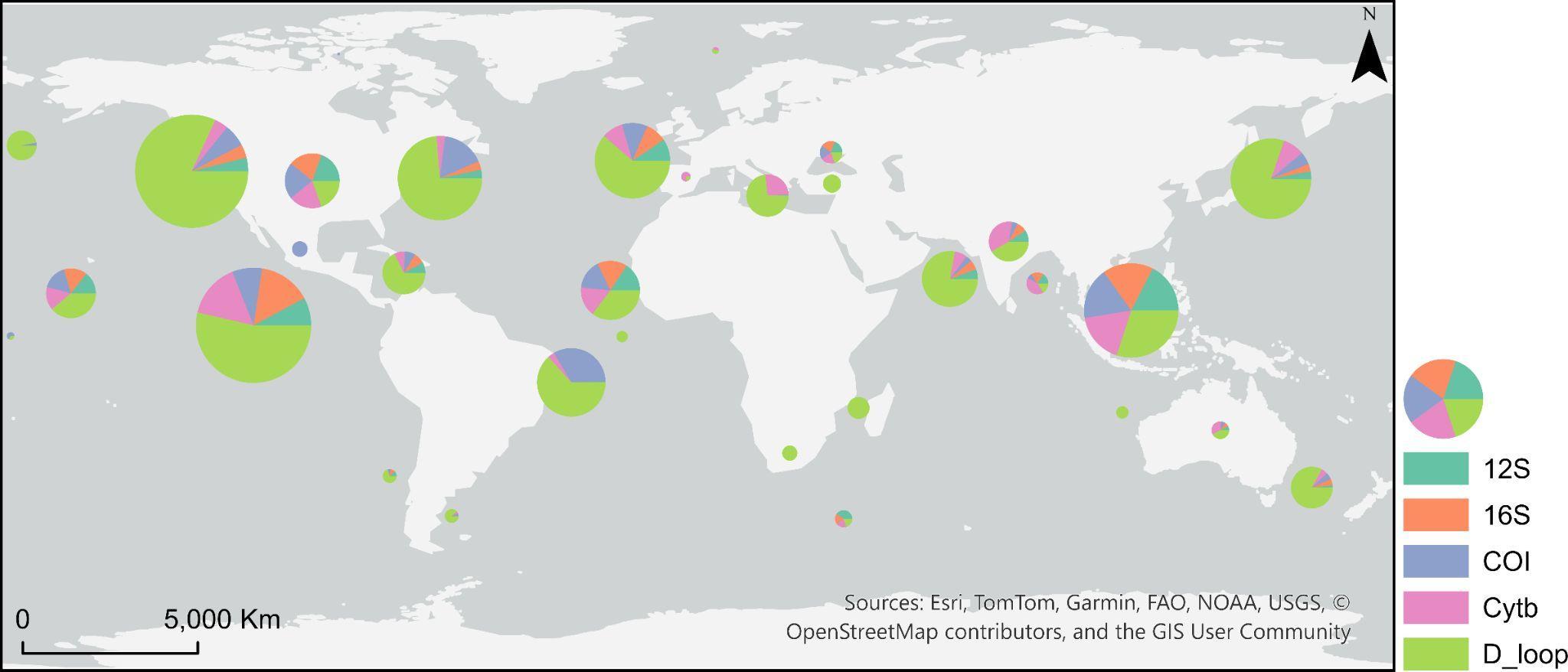
**

**D) Eschrichtiidae

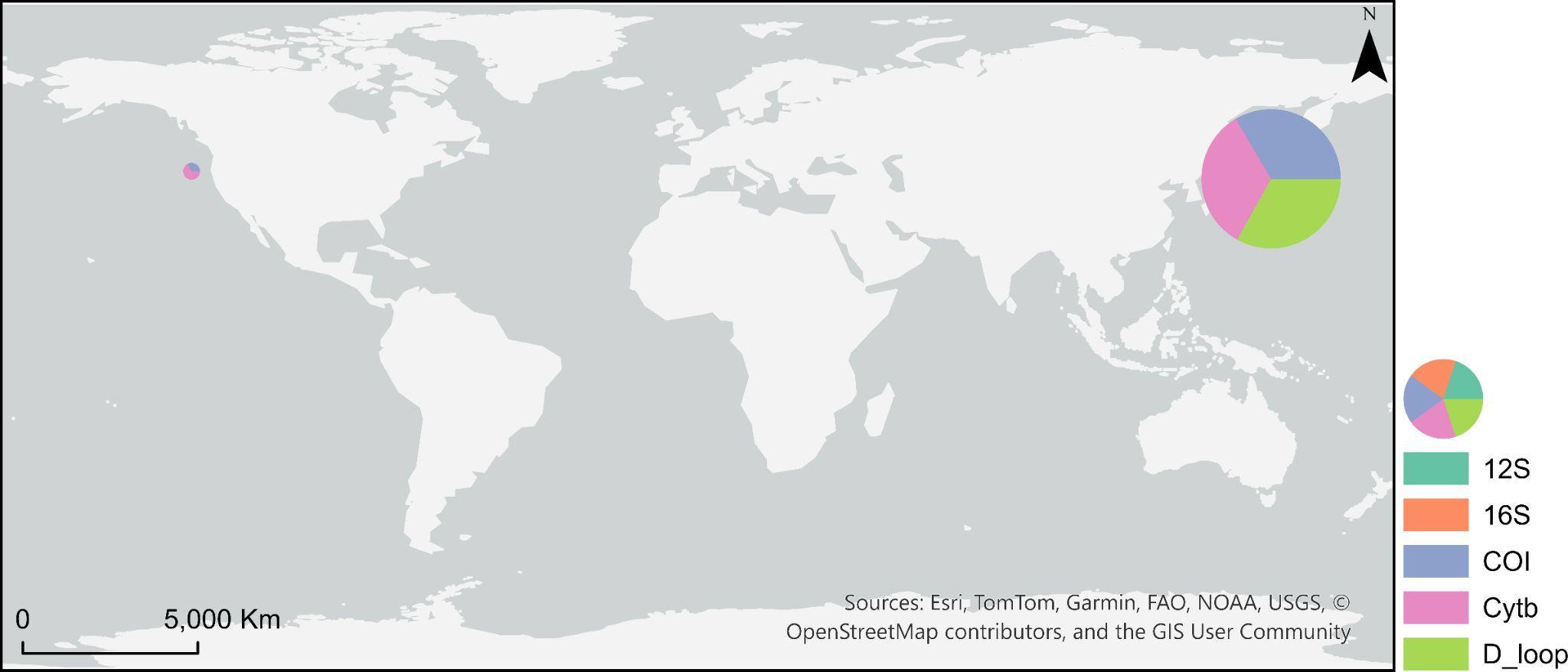
**

**E) Iniidae

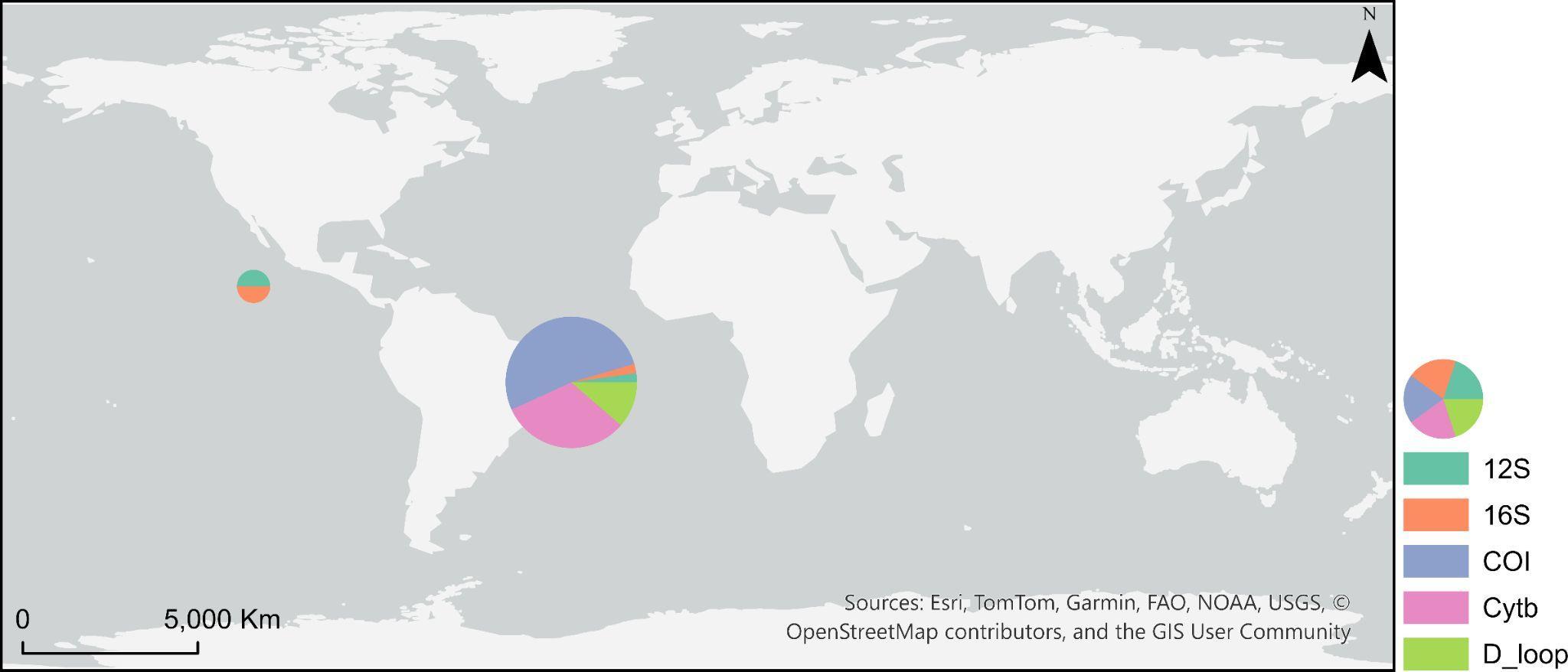
**

**F) Monodontidae

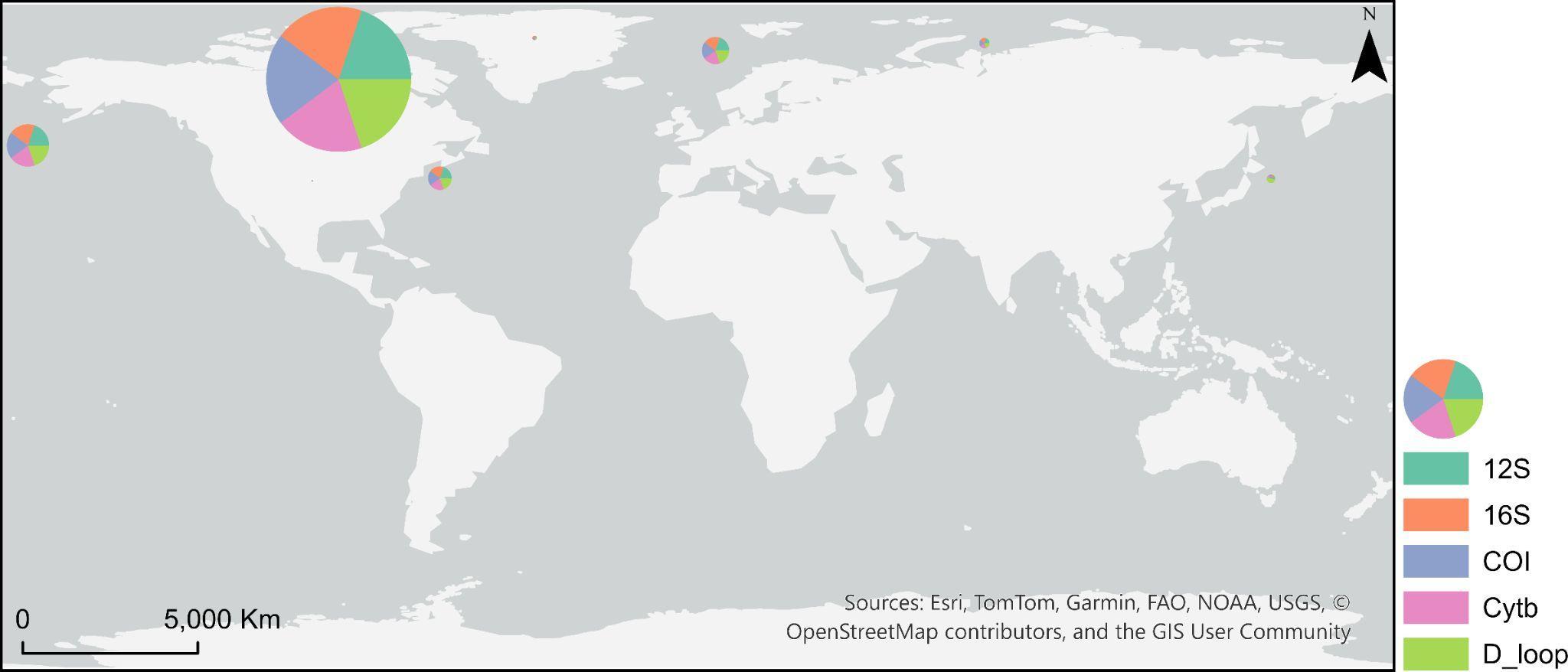
**

**G) Phocoenidae**

**
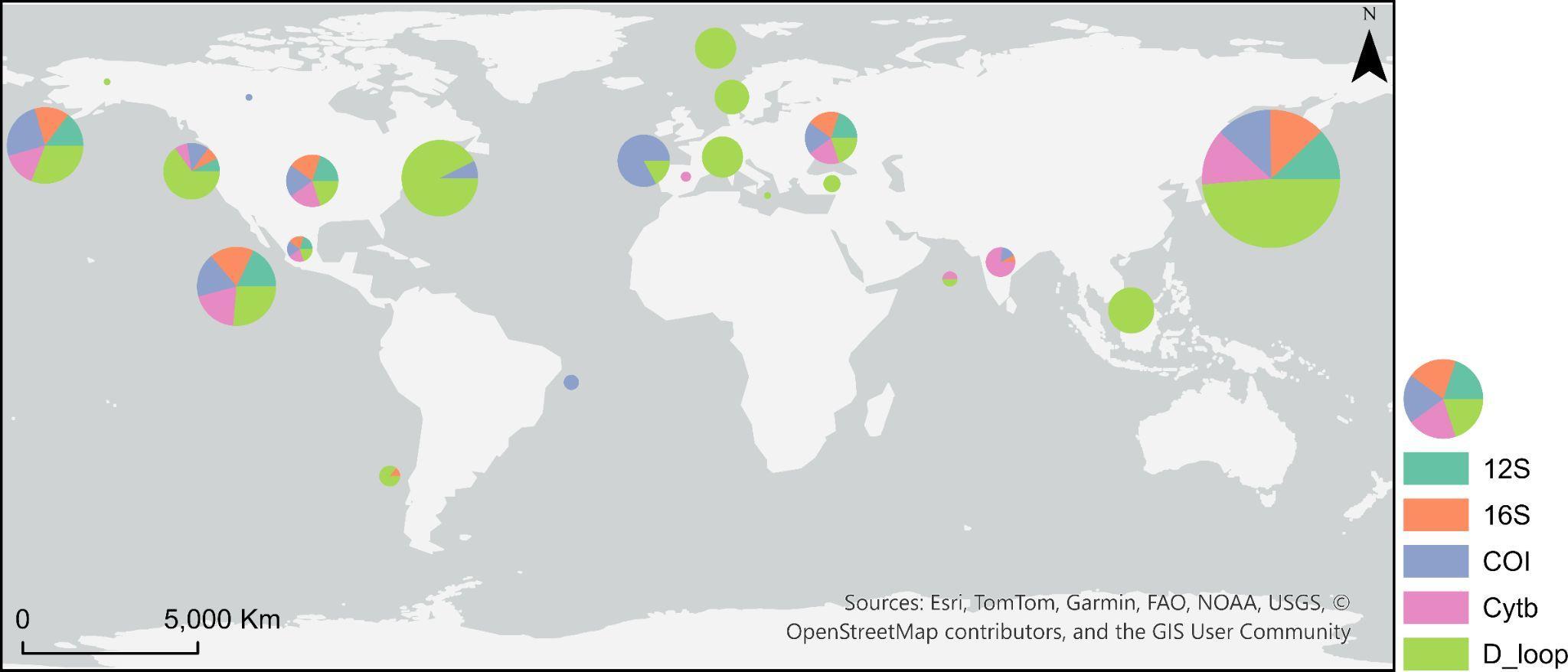
**

**H) Physeteridae**
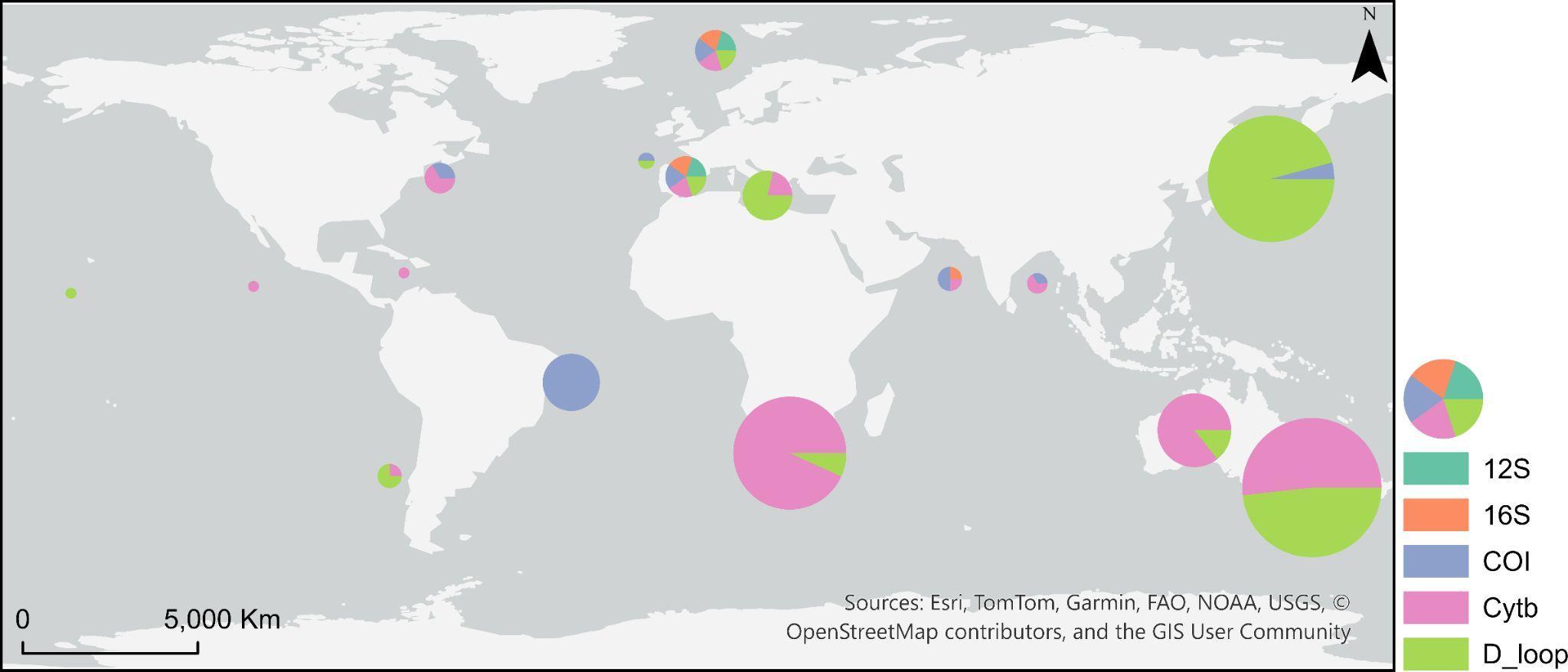


**I) Platanistidae

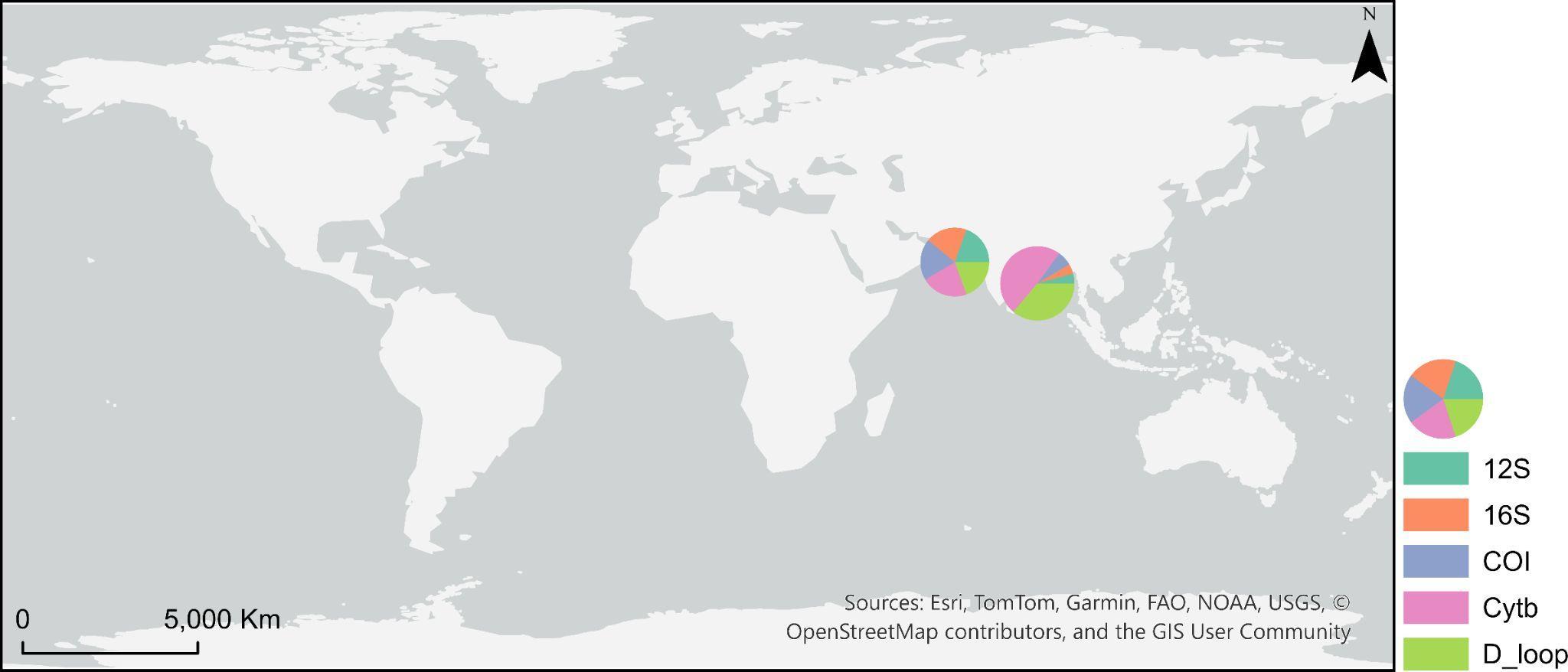
**

**J) Pontoporiidae

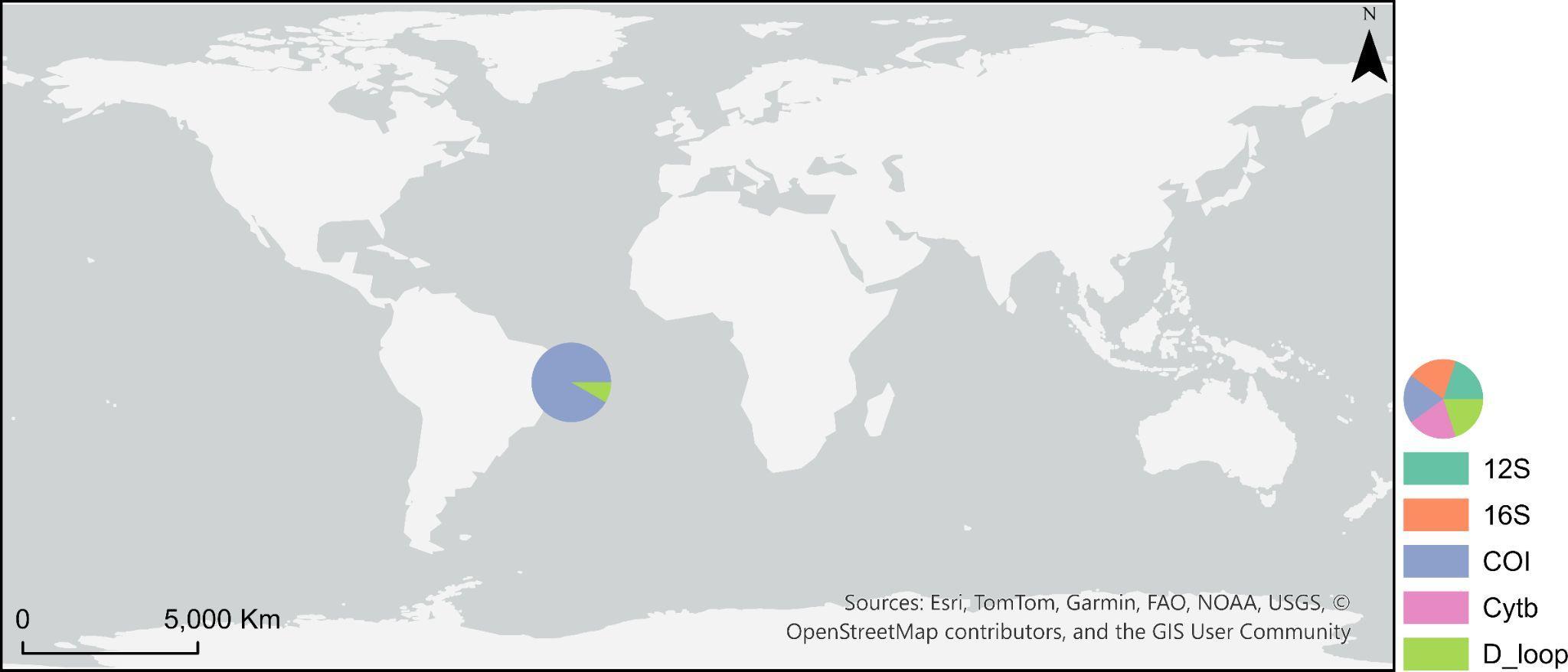
**

**K) Ziphiidae**


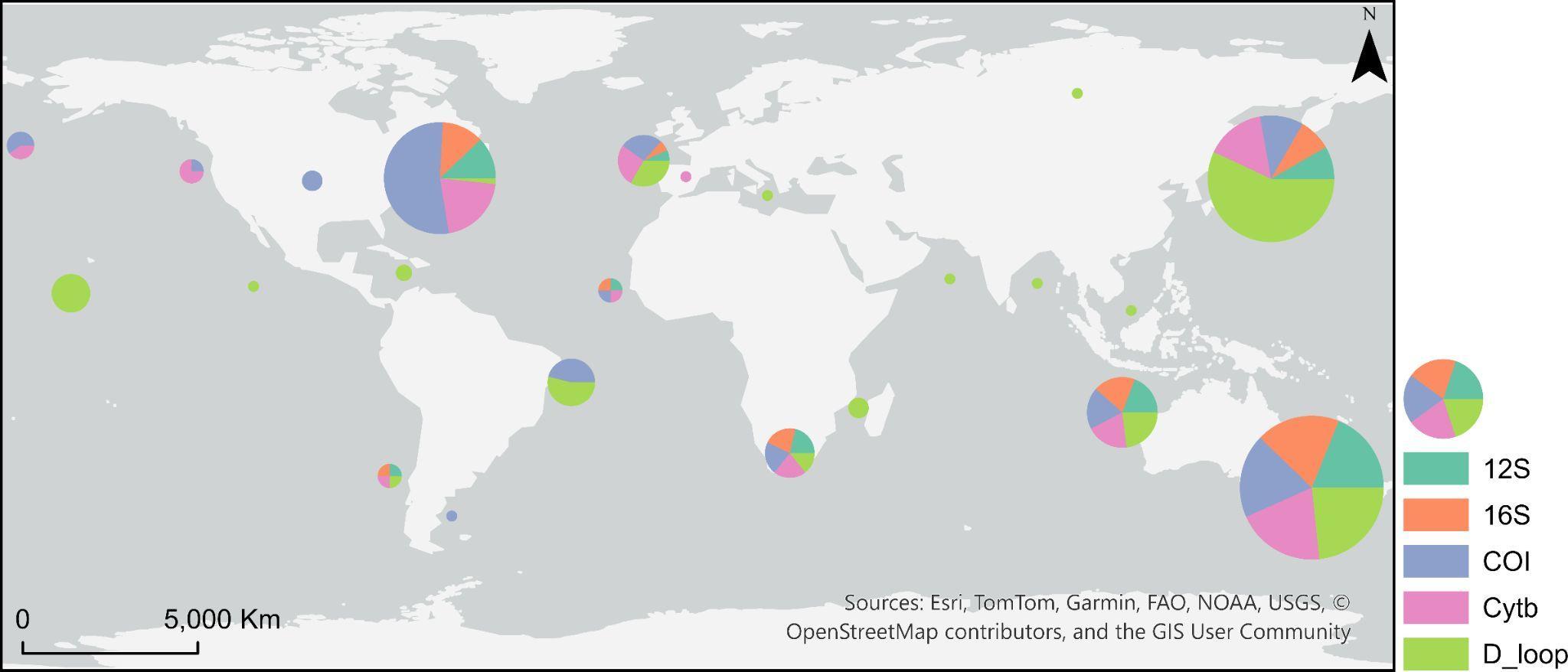

Supplement: Supplementary file 1 — Figure S1: (A, B, C, D). Marker abundance across cetacean species. Figure S2: Geographic distribution of cumulative sequences across cetacean families. [file MEN-26-e70141-s001.docx]
